# Beyond EDA: A Systematic Review of Multimodal Sympathetic Nervous System Arousal Classification for Stress Detection

**DOI:** 10.3390/s26051584

**Published:** 2026-03-03

**Authors:** Santiago Sosa, Adam K. Fontecchio, Evangelia G. Chrysikou, Jennifer S. Atchison

**Affiliations:** 1 Department of Electrical and Computer Engineering, Drexel University, Philadelphia, PA 19104, USA; ss5427@drexel.edu (S.S.); af63@drexel.edu (A.K.F.); 2 Department of Psychological and Brain Sciences, Drexel University, Philadelphia, PA 19104, USA; lilachrysikou@drexel.edu; 3 Department of Mechanical Engineering and Mechanics, Drexel University, Philadelphia, PA 19104, USA

**Keywords:** multimodal stress detection, electrodermal activity, nervous system arousal, wearable physiological sensing, sensor fusion, subject-independent modeling, deep learning, free-living stress assessment, affective computing

## Abstract

Electrodermal activity (EDA) is a powerful anchor for assessing human sympathetic nervous system (SNS) arousal. However, EDA alone is only one facet of physiological response. Researchers have increasingly moved away from single-sensor analysis to multimodal wearable systems, integrating EDA with other signals such as heart rate variability (HRV), photoplethysmography (PPG), skin temperature (SKT), blood oxygen (SpO_2_) and more. This critical shift in methodology is not yet reflected in current reviews of the literature. Existing surveys thoroughly cover EDA as a standalone measure, but the combination of sensor technologies has been largely unexamined. In this context, multimodal refers to integrating EDA with complementary biosignals (HRV, PPG, SKT, SpO_2_, etc.) commonly captured by modern wearable platforms. This review provides a comprehensive analysis focused on multimodal systems for assessing SNS arousal. A total of 58 studies met the inclusion criteria. We map the landscape, from single signal methods to complex sensor-fusion, and highlight advances in multimodal sensor models, physiological modeling, and context-aware sensing. We also examine recent advances in signal processing and machine learning that enhance multimodal SNS arousal inference, outlining current capabilities and identifying open directions for future work. By providing a framework of this emerging field, this paper serves as a resource for all researchers aiming to build and deploy the next generation of context-aware SNS arousal-sensing technology.

## 1. Introduction

### 1.1. The Physiology of Sympathetic Nervous System Arousal (Brief Recap: SNS/PNS, Cortisol vs. Fast Response)

To understand the engineering requirements of SNS arousal detection systems, one must first examine the physiological fundamentals of the autonomic nervous system (ANS), specifically the interplay between the sympathetic “fight-or-flight” response and parasympathetic regulation. The ANS functions as a control system that acts largely unconsciously and regulates bodily functions such as heart rate, digestion, respiratory rate, pupillary response, urination, and sexual arousal.

The ANS is divided into two primary branches that act in opposition to maintain homeostasis [1]:

- Sympathetic Nervous System (SNS): This system prepares the body for intense physical activity and is often referred to as the “fight-or-flight” response. When activated, physiological responses are triggered that create changes in heart rate, respiration rate, and sweat glands. Changes in sweat glands are assessed via electrodermal activity (EDA).
- Parasympathetic Nervous System (PNS): This system has almost the exact opposite effect, relaxing the body and inhibiting or slowing many high-energy functions, a state often described as “rest and digest”.

Modeling arousal requires capturing both SNS and PNS responses. In the context of SNS arousal, modeling is any computational/mathematical framework that maps signals to an inferred arousal state. Although EDA provides a direct measure of sympathetic arousal (sweat gland activity governed by the SNS), heart rate variability (HRV) provides a view primarily into the parasympathetic nervous system. Combining sensors that capture both systems allows for a broader overview of the state of an individual and a more complete reconstruction of the autonomic state that either signal can produce in isolation.

Physiologically, stress is not a single, uniform state and must be distinguished from related, but different concepts that also induce SNS arousal, such as cognitive load, excitement, physical exertion, and curiosity, among other states. Acute psychological stress refers to short-term physiological response to a stressor, such as public speaking or timed arithmetic, that disrupts homeostasis and activates the SNS. Mental workload is the measure of how much information must be processed to perform a task. While many workload and affective states can induce physiological responses, these measures primarily indicate sympathetic nervous system arousal, which may reflect stress, but also positive states such as engagement, anticipation, or joy. Stress is a contextually-specific interpretational state; that is, the individual assesses an experience to be stressful based on affect, appraisal, perceived demands and meaning in a specific situation. More so, long-term stress can also be present with low SNS arousal, in the form of burnout, depression, chronic worry, dread, etc. These are not associated with high SNS activity. Distinguishing and classifying the cause of arousal is crucial for accurate modeling of an individual’s state. Therefore, accurate SNS arousal detection systems must not only measure physiological signals, but also disambiguate correctly through computational psychometrics and detection algorithms [2].

### 1.2. The Shift from Unimodal to Multimodal

While Electrodermal Activity (EDA) serves as a sensitive indicator of sympathetic arousal, reliance on this single modality often results in ambiguity, prompting a paradigm shift toward multimodal systems that can triangulate arousal states with greater precision. EDA has long been used as a primary physiological marker of SNS arousal, because sweat gland activity is directly innervated by sympathetic fibers, EDA is highly sensitive to changes in arousal and has been widely adopted in laboratory stress studies. However, EDA is not specific to psychological stress. Many different human states or environments can influence arousal of the SNS, and hence increase sweat gland activity. Excitement, lying, and other emotional states can increase EDA levels and hence be mistaken as stress, but in reality are unrelated to the task at hand: measuring and classifying levels of stress. Overall, EDA measures arousal, not stress directly. The reliance on single signal systems of EDA alone can be fragile to external “free-living” factors such as physical movement, ambient temperature, and individual differences in skin properties. As a result, EDA alone can often lead to ambiguous interpretations of arousal states.

Empirical evidence has increasingly shown that no single physiological signal is sufficient to fully capture arousal across tasks, individuals, and environments. Akbar et al. conducted a systematic comparison of unobtrusive physiological sensors during a variety of computer-based tasks and found that EDA and heart rate, when measured in isolation, were inconsistent across participants and task types [3]. Their results show that wrist-based EDA and heart rate signals were susceptible to motion artifacts during computer use, limiting generalization for stress detection across tasks and individuals. Even when stress was induced, single-modality signals frequently failed to show consistent deviation from baseline across all subjects. These findings highlight the core limitation of unimodal approaches: any single channel is vulnerable to noise, context dependence, and task-specific interference.

This limitation only grows in real-world settings, where stress unfolds continuously and is embedded in everyday activities. Gjoreski et al. addressed this challenge by evaluating stress detection using wrist-worn devices in both laboratory and real-life settings [4]. Although they show that stress can be detected from wearable sensors, a key takeaway is that sensor performance improved substantially when multiple physiological signals were combined and interpreted in context. Multimodal models consistently outperformed unimodal signals, especially for real-life data, where physical activity and environmental variability introduce significant noise that impairs single-sensor reliability. Their results underline that physiological arousal signals often overlap between stress and non-stress states, and combining modalities allows for disambiguation.

Together, these studies highlight the inherent limits of unimodal EDA systems and underscore the potential of multimodal systems. Instead of treating stress as a unidimensional signal, multimodal approaches leverage the simultaneous manifestation of sympathetic arousal across multiple physiological signals, including electrodermal, cardiovascular, and respiratory metrics. By way of multiple signals, multimodal frameworks can improve accuracy and decrease ambiguity allowing for better generalization in real-world environments. This shift forms the foundation of modern stress classification systems and underpins the need for a systematic evaluation of multimodal SNS measurement strategies, which is the focus of this review.

Wearable psychophysiological measurement systems that combine electrodermal activity (EDA) and heart-rate variability (HRV) provide important complementary information to behavioral measures in the detection and characterization of stress, reflecting a long tradition in psychophysiology of using autonomic signals as implicit indicators of arousal and emotion regulation processes. EDA offers a direct index of sympathetic nervous system activation. In contrast, HRV (particularly its short-term, vagally-mediated components) captures parasympathetic control and autonomic flexibility, allowing researchers to assess physiological responses that may not be evident in overt behavior or self-report [5,6,7]. Classic work in this area from psychophysiology has shown that such measures can reveal covert arousal, effort, or load even when performance appears stable or when individuals lack introspective access or motivation to report their internal states [6,8]. In ambulatory and naturalistic contexts, the integration of EDA and HRV with behavioral markers (e.g., task performance, movement, or interaction patterns) has, thus, been argued to improve sensitivity to stress-related dynamics and enhance ecological validity [4,9,10]. At the same time, these physiological signals primarily index arousal and regulatory processes rather than affective valence. Elevated sympathetic activation or reduced HRV can therefore arise not only during stress, but also during non-stressful states such as excitement, engagement, or novelty detection. As a result, interpretation is strengthened by multimodal models that integrate physiological data with behavioral and contextual information, rather than treating autonomic measures as direct proxies for specific emotional states [7,10,11].

### 1.3. The “Context-Aware” Gap

Despite the proliferation of wearable sensors, a significant gap remains in translating laboratory-validated models into context-aware systems capable of robust operation in the noisy, dynamic environments of daily life. Many methods demonstrate high performance under controlled laboratory settings, but often degrade when deployed in daily life, where physiological signals are influenced by movement, environment, behavior, and individual variability. This presents not only an engineering limitation but also a challenge to model SNS arousal in context-dependent settings in order to determine whether arousal is due to stress or other factors.

Early work by Sano et al. represents an influential attempt to bridge this divide. The large-scale SNAPSHOT study combined multimodal wearable sensors with mobile phone data in order to study how stress and mental health are influenced in everyday life over the course of a month [12]. Their work moved beyond laboratory tasks and embraced real-world complexities. While their study demonstrates that multimodal features can predict self-reported stress with reasonable accuracy, the study highlights the difficulty of transition from lab to the real world. Continuous monitoring introduces noise at every level and requires extensive signal processing, quality control, and interpretation. Sano et al.’s work highlights the promise of context-aware stress monitoring and the substantial work needed to make such systems functional.

To further widen the gap, Nkurikiyeyezu et al. directly examined this issue and identified what is often left implicit: physiological stress models often fail when applied to new users [13]. Their analysis showed that models trained on EDA and HRV can achieve near-perfect performance when evaluated on known subjects but quickly collapse when tested on unseen individuals. This “gap” in generalization rises due to the fact that physiological responses to stress are highly individually specific. Baseline levels, ranges, and response patterns are highly variable across individuals, which makes universal modeling notoriously difficult. A model trained on one group may encode subject-specific signatures that do not translate to others.

Taken together, these findings expose a critical gap in the current literature. Multimodal sensing clearly outperforms unimodal methods, and early work has shown feasibility in natural settings. However, context awareness remains a largely unsolved issue. Existing studies often focus on specific datasets and subsets of individuals, leaving open questions of generalization and personalization. This review is therefore timely in that it focuses on multimodal SNS measurement through the lens of real-world applicability, with attention to context awareness and robustness of models, two requirements that can determine whether SNS arousal and classification systems move beyond prototypes.

### 1.4. Paper Organization and Scope

This review addresses these challenges by systematically mapping the multimodal sensor landscape, first by covering various kinds of sensors, beginning with EDA as the foundational sensor for SNS measurement. It then covers the integration of additional biosignals to support the transition from unimodal to multimodal sensing, with emphasis on sensor relevance and practical applicability. We review inferencing and modeling algorithms, dividing them into statistical/rule-based methods, classical machine learning methods, and deep learning methods. Finally, we examine context-awareness and robustness strategies that aim to close the gap between laboratory and real-world deployment.

A consideration throughout this review is the role of sensing hardware. Wearable devices vary significantly in signal quality, and physiological validity cannot be assumed by default. van Lier et al. showed that meaningful evaluation requires validation at the signal, parameter, and event levels, and wearable devices meet these criteria only under constrained conditions [14]. This “hardware issue” places a limit on stress classification performance.

Accordingly, this review distinguishes between systems built on validated physiological measurements (such as the WESAD or ADARP dataset) and those that rely on consumer-grade devices. WESAD (Wearable Stress and Affect Detection) is a widely used benchmark dataset for multimodal stress detection, providing synchronized physiological signals (EDA, ECG, EMG, respiration, and temperature) collected under controlled stress and baseline conditions with standardized protocols, enabling reproducible comparison across studies. ADARP (Alcohol and Drug Abuse Reseach Program) is similarly adopted for evaluating generalization and subject-independent performance because it includes multimodal wearable recordings with rigorous labeling and is frequently used for leave-one-subject-out validation to test real-world robustness. We aim to provide a realistic assessment of current multimodal SNS arousal classification methods and inferencing algorithms, along with providing clarification of where reported gains reflect genuine advances versus hardware-dependent effects.

## 2. Methodology

### 2.1. Search Strategy

To ensure a comprehensive and unbiased review of the current literature on multimodal arousal detection, a literature search was conducted using the following databases: IEEE Xplore, PubMed, Scopus, and Web of Science, along with preprint repositories (arXiv, medRxiv, and bioRxiv). The search covered all papers from 2020 to 2025, capturing the current state of the art and the fast pace of change in sensor systems. A small number of earlier studies were included which represent foundational work or widely cited benchmarks necessary for contextualizing later advances. No protocol was registered for this systematic review, and no formal review protocol was prepared prior to conducting the study.

To search for relevant studies and maintain reproducibility, queries utilized Boolean operators to combine key terms related to EDA, sensor fusion, arousal, and inference/modeling. The primary strings included are shown in Table 1.

### 2.2. Inclusion and Exclusion Criteria

The search method produced a total of n = 1287 articles across all databases. After removing duplicates, n = 809 remained. Zotero 7.0.32 software was used to automatically identify duplicate entries and supported preliminary exclusion of clearly ineligible records before manual screening. The remaining studies were then screened by title and abstract to refine the focus of the review. This was done by determining the exclusion criteria as follows:

- Non-matching Topic: Papers focusing on epilepsy, seizures, pain, lying/deception, general emotion recognition, or sleep. These papers do not focus on direct detection of arousal or stress, and thus were removed.
- Non-matching Sensors: Papers that focus heavily on EEG, fMRI/MEG, biomarkers (cortisol, etc.), or other non-wearable/single-signal sensors. These papers do not focus on wearable multimodal systems, and thus were removed.
- Non-matching Technology: Papers that focused on stationary equipment that is not deemed wearable, or simulation-only data.

**Figure 1:**
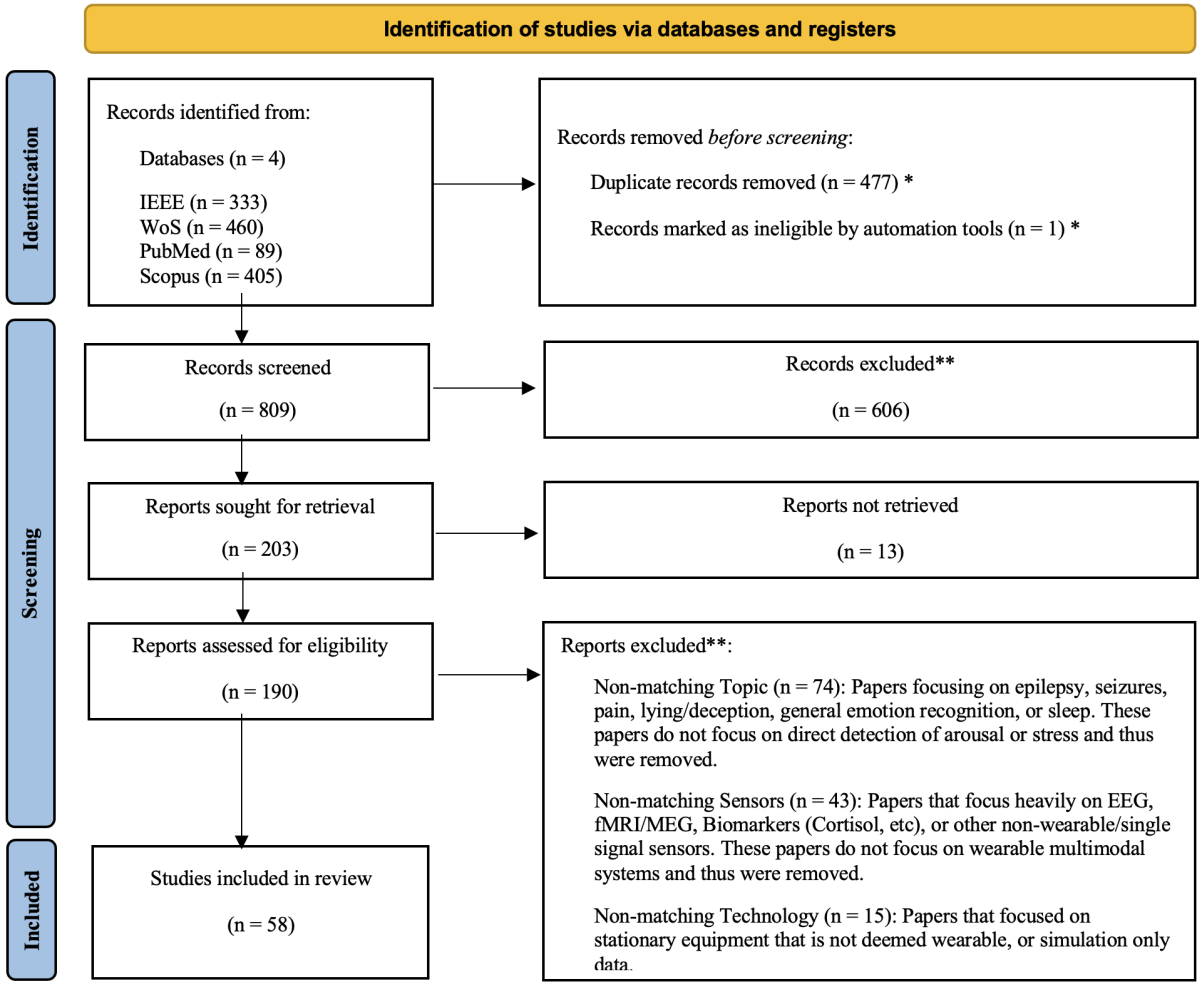
Inclusion/Exclusion criteria methodology based on PRISMA screening procedure. * Reference management and deduplication were performed using Zotero 7.0.32, which automatically identified duplicate entries and supported preliminary exclusion of clearly ineligible records before manual screening. ** The same exclusion criteria were applied during both the “Records screened” and “Reports assessed for eligibility” stages of the PRISMA screening process.

By the end, n = 58 papers remained. The PRISMA screening procedure is illustrated in Figure 1.

### 2.3. Data Extraction and Classification

Selected papers were “binned” into categories based on four main overarching themes:

- The Multimodal Sensor Landscape
- Architectures of Sensor Fusion
- Inferencing and Modeling Algorithms
- Context Awareness and Robustness

**Figure 2:**
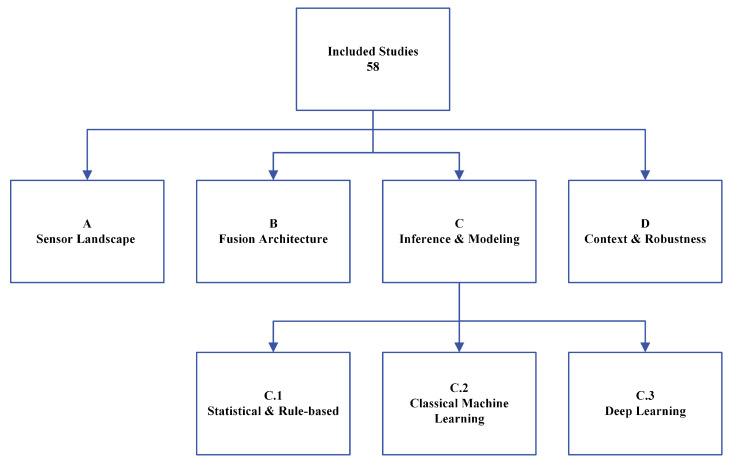
Illustrates the organization of the papers cited in this review according to four main themes.

Figure 2 illustrates the subdivision of the papers into the four themes, theme C (Inferencing and Modeling) is then subdivided into 3 subthemes reviewing the 3 main computational approaches for SNS arousal inferencing and modeling: (1) Statistical and Rule-Based Methods, (2) Classical Machine Learning Methods, and (3) Deep Learning Methods. These “buckets” map directly onto the organization of the review, each of the following sections mainly covering one bucket. Each paper was assigned one primary theme, but was not exclusive to that bucket. If a paper substantively contributed to another theme, one or more themes were also assigned as “secondary” themes, mainly defined by explicit content relevant to those themes (e.g., testing various Classical Machine Learning algorithms against Deep Learning models). Fusion architectures were also noted, depending on the approach taken by the paper. This is further explained in Section 4. A table summarizing all included studies is provided in Table 2, including modality codes, fusion strategy, primary model used, and primary result. Fusion strategies were classified as follows:

- Unimodal: Single sensing modality used for inference (no fusion).
- Data/Early Fusion: Data from sensors are combined at the input level before feature extraction or modeling.
- Feature/Intermediate Fusion: Features are extracted or handcrafted from sensors separately, then combined for modeling.
- Decision/Late Fusion: Separate models are trained on each sensor, and their outputs are combined for final inference.
- Compared: Multiple fusion strategies are directly compared within the same study to evaluate their relative performance.
- Hybrid: A combination of the above fusion strategies is used within the same model architecture, such as using both feature and decision fusion in different stages of the model.

### 2.4. Risk of Bias Considerations

Risk of bias varied across included studies and was evaluated qualitatively during synthesis. While most papers reported general sensor configurations and modeling pipelines, transparency regarding preprocessing steps, artifact handling, and signal-quality filtering was not consistent. Sample sizes were frequently modest, and demographic reporting varied in completeness, limiting assessment of population representativeness. Definitions of stress or arousal ground truth differed substantially across datasets, ranging from controlled laboratory protocols to self-reported or context-derived labels, introducing heterogeneity in evaluation targets. Particular attention was given to dataset dependence (e.g., frequent reliance on WESAD), which is discussed further in Section 7.2. Model validation procedures were also variable. Several studies did not clearly specify subject-independent testing or separation between training and evaluation cohorts. These factors were considered when interpreting reported performance metrics and when comparing results across studies.

A qualitative risk-of-bias assessment was performed to evaluate methodological transparency and potential sources of systematic error across included studies. Because the reviewed literature spans wearable sensing, signal processing, and machine learning-based stress inference, traditional clinical bias tools were not directly applicable. Instead, studies were evaluated descriptively with respect to participant reporting, sensor specification and preprocessing transparency, validity of stress labeling or ground truth, clarity of model validation procedures (including subject-independent testing), and completeness of performance reporting. Risk-of-bias considerations were incorporated into the narrative synthesis rather than used as exclusion criteria, and methodological limitations were considered when interpreting reported performance metrics.

## 3. The Multimodal Sensor Landscape

### 3.1. The Anchor: EDA as the Primary Indicator of Sympathetic Arousal

Electrodermal Activity (EDA) remains the cornerstone of modern SNS arousal detection architectures, used for detecting stress, due to its direct and linear correlation with the sympathetic nervous system, and serving as the primary “anchor” for detecting acute arousal events. When arousal of the sympathetic nervous system occurs, activity increases in the sweat glands. Due to the conductivity patterns of sweat, the conductance of the skin at the surface changes accordingly (typically measured in microsiemens (μS)). In practice, EDA is decomposed into two separate components, slow baseline and fast event-like bursts. This split maps onto how sympathetic nervous system arousal is manifested as skin conductance [66].

- Tonic Component (SCL: Skin Conductance Level):
  The tonic signal shows as the background level of skin conductance, with a slow drift over time (tens of seconds to minutes), capturing the baseline level, gradual ramping, recovery, and longer trends. Regarding features, the tonic component is what provides the mean/median SCL, slope, variance, range, baseline shifts, and recovery trajectories.
- Phasic Component (SCR: Skin Conductance Response):
  The phasic signal is a transient response, short bursts that occur when sympathetic drive produces rapid sweat gland activity. These appear as peaks with a rise and decay, typically lasting a few seconds. Regarding features, phasic allows for peak count (event rate), peak amplitude, rise time, recovery time, and inter-peak timing. From an engineering perspective, frequency analysis allows for capturing differences in stress versus non-stress conditions.

**Figure 3:**
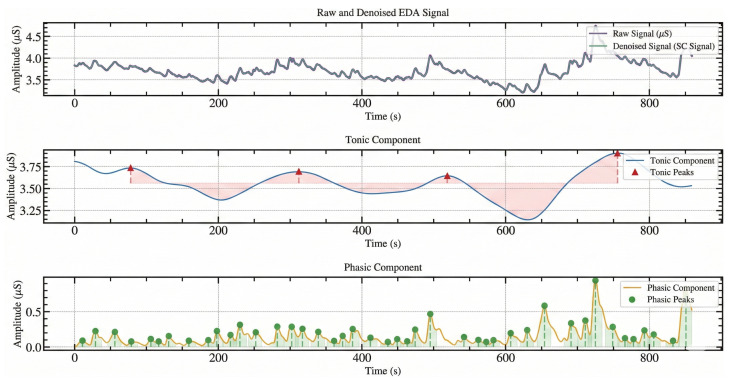
Denoising and decomposition of EDA signal: visualization of raw signal, denoised output, and tonic and phasic components. Adapted from [45]. Licensed under CC BY 4.0.

The phasic component of electrodermal activity (EDA) captures transient, event-related skin conductance responses (SCRs) driven by sympathetic nervous system activation, as shown in the Figure 3 phasic component graph [67]. The phasic component of the EDA signal is sensitive to both cognitive load and emotional salience, though these influences manifest differently in the signal. Increases in SCR frequency and moderate amplitude changes are commonly associated with heightened cognitive effort, sustained attention, and task engagement, whereas emotionally salient or aversive stimuli tend to elicit larger-amplitude SCRs, reflecting stronger autonomic arousal [66]. Notably, “aha moments”, or insight events which combine sudden cognitive restructuring with an affective component of surprise or reward, often produce distinct, time-locked phasic responses, making SCR amplitude and timing useful markers for identifying moments of insight during problem solving [68]. As such, phasic EDA provides interpretable information about the timing, intensity, and qualitative nature of cognitive–emotional events, supporting its use as an anchor signal in studies of cognition, emotion, and creative problem solving.

This makes EDA a strong method for detecting acute arousal, as peaks and shifts in both the tonic (SCL) and phasic (SCR) signals are often visible, while other biosignals can appear subtle or noisy. Figure 3 shows the decomposition of an EDA signal, visualizing the tonic and phasic components, reproduced from [45]. EDA alone does not “solve” the classification of SNS arousal, but it provides a grounded reference signal that when integrated with other biosignals, can contextualize the arousal state of an individual.

This frame is strongly supported by modality comparison work that answers the following question:

If you had to pick one biosignal, which one gives the most accurate classification and estimation of stress?

Holder et al. evaluated both multimodal and single-modality stress classification using common biosignals (acceleration, blood pressure volume, EDA, and skin temperature). They found that while multimodal combinations can yield strong performance, EDA consistently emerges as the most reliable standalone signal for stress classification and generalization [15]. In fact, Holder et al. show that from raw data on a Convolutional Neural Network (CNN), EDA has an accuracy of 0.9209 on WESAD, comparable to the 0.9835 of all modalities combined on a 3/4-1/4 validation of a WESAD dataset. This strengthens the argument that electrodermal activity is an anchor signal for SNS arousal detection. Holder et al. achieved these results by utilizing raw accelerometer data sampled at 32 Hz and segmented into one-second sliding windows with 50% overlap, allowing the model to leverage the full temporal resolution of the signal without the downsampling required for multimodal fusion. Accelerometer data performed similarly, with 0.9587 on the K-Nearest Neighbor (KNN) classifier, but can be much more context dependent. Holder also performed leave-one-subject-out (LOSO) validation on an ADARP dataset, and found that EDA has the highest accuracy (0.7479), but a low F1-Score (0.0809) compared to all modalities combined (accuracy: 0.4952, F1-Score: 0.3402). This shows that EDA is a very strong signal, but struggled alone with generalization to unseen subjects.

However, EDA’s role as the default anchor must be qualified by evidence showing that its standalone discriminative power is not universal across tasks, populations, or sensor configurations. Holder et al. themselves illustrate this: while unimodal acceleration achieved 0.9587 accuracy on the WESAD dataset (3/4-1/4 validation), EDA alone yielded only approximately 0.9199, a case in which EDA raw data showed lower performance relative to another modality [15]. This finding suggests that EDA alone does not guarantee improvement when choosing signals, and that the choice of complementary modality matters as much as the decision to fuse. In a different domain, Kalimeri and Saitis found that EDA achieved an AUROC (Area Under the Receiver Operating Characteristic) of only 0.533 during indoor mobility tasks compared to 0.773 for EEG. AUROC is a performance metric for binary classification, which represents the probability that a model ranks a random positive example higher than a random negative one. Ranging from 0.5 (random) to 1.0 (perfect). Fusion of EDA with EEG improved AUROC modestly to 0.793. In the study, the performance was clearly dominated by the neural signal rather than the electrodermal one [23]. These findings do not invalidate EDA as a useful measure of sympathetic arousal, but they do indicate that its reliability as a standalone classifier is task dependent and population dependent. In contexts involving physical movement, cognitively driven stressors, or populations with low electrodermal responsiveness, EDA may contribute less discriminative information than cardiac, respiratory, or neural signals. The “anchor” framing should therefore be understood as a physiological claim—EDA directly indexes sympathetic sweat gland activity—rather than an engineering guarantee that it will outperform other modalities in every classification pipeline.

Recent wearable devices demonstrate a common pattern where EDA is the primary indicator used to identify SNS arousal; however, other signals such as cardiovascular signals can be used to add supporting evidence and refine signal analysis. Subathra et al. implemented a low-cost wearable band that pairs EDA, measured via galvanic skin response (GSR) and captured as skin conductance responses (SCRs) in the phasic component of the signal, with PPG-derived cardiac metrics, using interbeat intervals (IBI) as core features for stress inference [56]. IBI is the exact measure (in milliseconds) of time between heartbeats. Subathra et al.’s pipeline uses the “EDA Anchor” concept practically, decomposing EDA into tonic/phasic structures (via SCL) and incorporating cardiac timing features to provide a second signal that is sensitive to sympathetic arousal. The implication for the wearable sensor landscape is straightforward: as architectures move toward multimodal fusion, EDA remains the central reference on which stress inference is built.

### 3.2. Cardiac Integration (EDA + HR/HRV)

The integration of cardiac metrics such as Heart Rate Variability (HRV) with EDA has emerged as the foundational pairing in multimodal systems, offering a dual perspective that captures both the intensity of arousal and the valence of emotional response. HRV is often measured from ECG (electrical R-R intervals) or from wrist PPG signals (blood volume pulse, used as a proxy for beat-to-beat timing). Heart rate captures the “how fast” of the cardiac response and HRV captures the “how variable the response is”, and that variability carries information about autonomic regulation, not just the magnitude of arousal. This is why EDA + cardiac is a strong pairing. EDA provides a window into sympathetic arousal and HRV reflects how the heart is modulated by both the sympathetic drive and parasympathetic (vagal) control [51].

The complementarity of these measurements is evident when one considers what each signal is missing. EDA, although excellent at flagging that something is activated in the SNS, does not reliably portray what that arousal is (e.g., stress, physical effort, excitement, etc.). Cardiac signals provide complementary information, as arousal-related states modulate heart rate and heart rate variability through changes in autonomic balance, particularly vagally mediated components that withdraw under load and rebound during recovery. In controlled settings, simple models may rank cardiac-derived features as competitive predictors relative to skin conductance; however, these signals primarily reflect regulation and recovery dynamics rather than the emotional valence of the eliciting event. Accordingly, combining cardiac and electrodermal features improves sensitivity to autonomic state but does not, on its own, resolve whether arousal reflects stress, excitement, or other affective conditions. Iqbal et al. tested this and reported better predictive value for respiration/cardiac variables than EDA in their dataset, and noted that a compact combination (respiration + HR + HRV) can achieve similar classification performance to using all measured modalities while being implementable in a single PPG-style sensor [33]. Iqbal’s work shows that respiration alone achieves an accuracy of 0.7696, but by adding heart-rate with respiration, the model’s accuracy increases to 0.8281. When adding all six modalities used in the study, there is marginal improvement to 0.8571. This shows the significance that cardiac features can have on SNS arousal classification and detection. Other studies also show improvements from cardiac features, such as Arsalan & Majid in their study of stress during public speaking. The group ran classical machine learning models on a combination of biosignals. The accuracy of only EDA and EEG was 0.85; however, by adding PPG into the model with the other modalities the accuracy increased to 0.9625 [31].

From an engineering perspective, pairing cardiac-derived metrics depends strongly on how they are calculated and interpreted. HRV, for example, has a family of features on its own, and which you choose can have large impacts. Time-domain metrics (such as RMSSD—Root Mean Square of Successive Differences) are easy computationally and broadly used in the literature, but can miss detail that would show in the frequency or time-frequency domains. Boffet et al. showed that classical HRV markers (such as RMSSD and Fourier-derived bands) do not reliably separate cognitive workload from emotion induction conditions. However, time-frequency processing (via VFCDM/WPT HF markers) revealed complementary autonomic dynamics, with the strongest predictive performance emerging from pairing a sympathetic EDA marker ($EDA_{TVSYMP}$) with a vagal HRV marker ($HF_{VFCDM}$). Importantly, this improvement reflects sensitivity to autonomic regulation and recovery dynamics rather than emotional valence. They also investigated whether participants could be classified in clusters of “autonomic profiles”, observing that some individuals express load more through EDA and others more through HRV. So reliance on single modalities can be unreliable on a large scale [51].

Contemporary papers have built upon those pipelines (EDA + HRV) and reached similar conclusions: mean levels are often insufficient, and signals are carried by dynamic features. Al Fawwaz, for example, showed that mean EDA differences across workload can be detectable but modest in effect, whereas event-like measures (like peak count after tonic/phasic decomposition) are more discriminative and embed those EDA dynamics along HRV features in a unified workload classification workflow [45]. That provides further support for EDA + HRV as a default pairing. Mathur et al. have also found significance in the multimodality pairings, as they determined the F1-Score of HRV to be the best in terms of unimodal systems (0.79 and EDA of 0.63), but multimodal systems achieved a 0.99 F1-Score, with all tested on various classical machine learning algorithms (KNN, DT, RF, etc.) [48]. Sympathetic bursts and drifts from baseline are detectable from skin conductance, and autonomic regulation/recovery can be derived from the heart. Jointly, these models are stronger than either one individually.

### 3.3. Thermal and Respiratory Integration: Improving Robustness in Physical Activity

To enhance robustness against environmental confounding variables, researchers have increasingly incorporated skin temperature and respiration rates, which provide critical context for distinguishing SNS arousal from thermal regulation or physical exertion. Once hardware leaves the laboratory, failure modes often manifest as false detection of stress. The body can appear stressed for reasons that are not psychological. Heat, movement, posture, and breathing all play a role in this. EDA and cardiac metrics are sensitive to sympathetic arousal but are also sensitive to thermal regulation and physical activity, creating a confound in which models detect arousal while lacking the contextual information needed to distinguish psychological stress from physiological demand.

Adding skin temperature (SKT) and respiration/breathing rate (BR) has contributed to a solution toward filling in the missing details. These signals provide context that can help separate stress from movement or thermal regulation. Akbar et al. demonstrated the problem in a real setting by measuring EDA/PPG on the wrist, ECG/BR on the chest, and a thermal imaging-derived perspiration signal across multiple computer tasks [3]. The results showed that wrist EDA and wrist HR were not always consistent for task discrimination, whereas respiration showed more generalizable sensitivity to task-induced stress. They also explored wearable performance and motion artifacts during typing and task-dependent interference (e.g., speech affects breathing rate). The conclusion was not that EDA is counterproductive, but that EDA + HR can be fragile, and adding SKT and BR channels can increase the robustness of models by providing information on physiological changes induced by non-stress tasks.

### 3.4. Emerging Modalities: Accelerometry and SpO_2_

Recent innovations in wearable form factors have expanded the sensor landscape to include optical SpO_2_ measurements and ear-based sensors (hearables), opening new avenues for unobtrusive, continuous monitoring. As the domain matures, more channels that were once treated as a second thought have now become crucial for contextualization of arousal. Two primary examples are accelerometry (ACC) and optical sensing beyond simply heart rate. The common theme is that these modalities help keep arousal detection and classification reliable when the sensor environment becomes unstable (such as during movement, or for alternative sensor placement).

Accelerometry is the key to solving one of wearable technology’s strongest adversaries: motion. Motion not only adds noise but can displace sensors and elevate metrics such as HR or EDA, even in activities as simple as getting off the couch. Rahman et al. studied ways to measure multiple metrics through the ear, including ACC, HR, and SKT, and found that these channels can be used to handle real-world artifacts and extract additional physiological signals. For instance, respiration features can be derived from the earbud accelerometer. In their pipeline, segments of poor wearable signal quality were explicitly identified (e.g., poor sensor fit or excessive head motion), and multimodal sets (HR/HRV + BR + SKT) outperformed narrower cardiac-only baselines in stress classification [39]. It is important to note that accelerometry does not simply offer measures of activity, but can also serve as a signal quality and context sensor.

**Figure 4:**
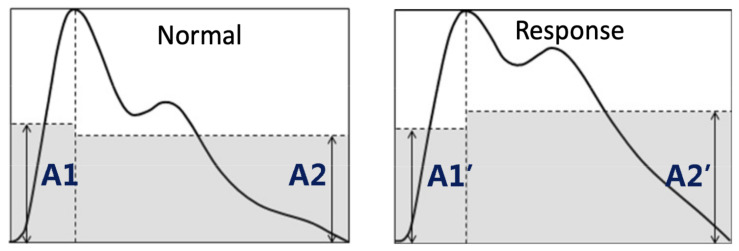
Schematic illustration of photoplethysmogram (PPG) waveform morphology under baseline and task-evoked response conditions. The stress-induced vascular response is quantified by changes in the relative amplitudes of two waveform contours (A1 and A2), which shift to A1′ and A2′ under increased sympathetic activation. This contour-based ratio forms the basis of the stress-induced vascular response index (sVRI). Reproduced from [63].

Additionally, optical sensing can provide more sophisticated measures of vascular response, which heart rate alone cannot capture. Specifically, optical methods can provide measurements of blood oxygen levels (SpO_2_). PPG is already a pillar of pulse oximetry, and Lyu et al. emphasized that motion-robust PPG processing is a well-studied problem in oximeters, which is relevant as stress monitoring leaves the lab [63]. Moreover, the group demonstrated that PPG may capture stress-relevant information regarding sympathetic arousal, as it drives peripheral vasoconstriction, the narrowing of small blood vessels. This directly alters the waveform measured. Their proposed stress-induced vascular response index (sVRI) uses a ratio between two waveform contour amplitudes (A2/A1) specifically to reduce the individual differences that create instability. They presented an algorithmic framework (pattern extraction + dynamic windowing + statistical moderation) designed for reliable, near real-time updating. See Figure 4 for illustration of the PPG waveform morphology changes under stress [63].

The wrist is not the only stable mounting point; the chest, ear, feet, and hands all have their roles. Rahman et al., for instance, showed that commodity earbuds can passively collect ear-PPG, inertial data, and body temperature, and even classify stress responses with subject-independent evaluation, while explicitly discussing hardware constraints like PPG sampling rate that can affect HRV measurements [39]. Based on these findings, the recommendation is not to simply add more sensors but to add sensors that keep inference valid in real-world contexts.

**Figure 5:**
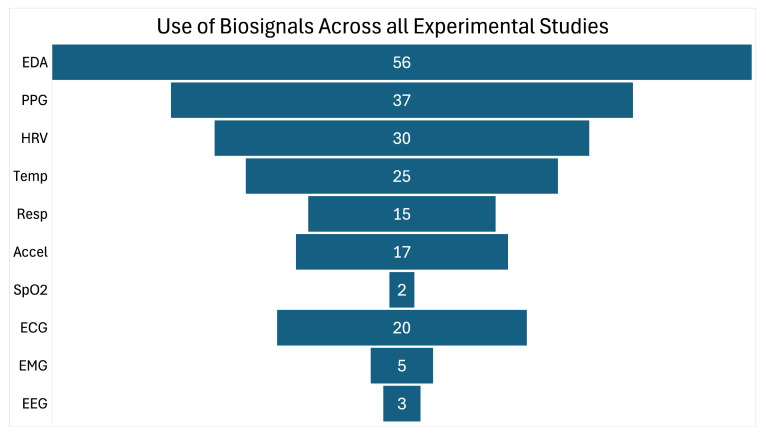
This figure illustrates the use of biosignals across all experimental studies reviewed in this paper, substantiating the claim that EDA serves as an anchor biosignal.

Modalities such as electroencephalography (EEG) and electromyography (EMG) have also appeared in the literature of stress classification. Both methods allow for a view into other parts of the nervous system, with EEG accessing the central nervous system (CNS) and EMG offering the opportunity to record from the somatic nervous system. EEG is often recognized as a “gold standard” of neurophysiological signals, giving researchers the ability to directly trace cognitive engagement and response inhibition, which peripheral signals may miss [23,48,63]. Complementing this technique, EMG serves as a sensitive indicator of muscle tension and skeletal activity, providing an objective metric for the physical “bracing” or restlessness that frequently accompany acute psychological stressors [33,42]. When combined with EDA, EMG can help separate cognitive effort from generalized arousal-related physiological activation, though this combination does not distinguish the emotional valence of the eliciting state [23,42]. However, the transition from laboratory equipment to real-world measurement of EEG and EMG has been a hardware obtrusiveness challenge. Though non-invasive, the requirement for multi-electrode arrays and skin preparation presents a significant usability barrier compared to ubiquitous wrist-worn platforms [49,54]. Various modalities can be selected based on the application requirements, but EDA remains the anchor signal across all reviewed studies, as shown in Figure 5.

## 4. Architectures of Sensor Fusion

Though there are three main levels of fusion (data, feature, and decision), some papers were categorized as “compared” as they directly compared the performance of different fusion levels rather than proposing a single architecture. The following sections will discuss the three main levels of fusion which are often compared. Furthermore, “Hybrid” models are becoming increasingly common, as they combine multiple levels of fusion to mitigate the costs of singular fusion levels. However, there is often one dominating fusion stage of the three covered below.

### 4.1. Data-Level (Early) Fusion: Granular and Raw Signal Approach

Data-level fusion represents the most granular approach to integration, attempting to combine raw signal streams at the input stage, though this method faces significant implementation challenges regarding temporal synchronization and disparate sampling rates. There are two major synchronization problems:

- Sampling-rate mismatch: Wearable systems often sample modalities at different rates (e.g., low-rate EDA stream versus high-rate PPG stream). If you concatenate raw samples, you implicitly force one signal to be downsampled, upsampled, or interpolated to match the other. This can distort exact structures of signals that must be preserved, especially in EDA where meaningful dynamics unfold over seconds whereas PPG deals with millisecond timescales.
- Physiological asynchrony: Even if two signals are sampled at the same rate, their response to the sympathetic nervous system can be different. One may occur at a delay with respect to the other. Sweat glands (EDA) and cardiovascular dynamics (PPG) reflect SNS activation through different pathways and different time constraints, so peaks and transitions do not directly line up on the temporal scale. Treating them as perfectly aligned injects errors into the fusion step and forces models to learn about mis-specified assumptions.

Beyond these two failure modes, early fusion is only well-defined if the pipeline specifies an alignment rule. In practice, studies rely on three simple strategies. First, resampling: all streams are mapped to a common time grid, which makes concatenation easy but can distort signals when one modality is naturally slow (EDA) and another is fast (PPG). Second, window-based alignment: signals are segmented into fixed-length (or sliding) windows and fused at the window boundary rather than sample-by-sample. This avoids strict pointwise matching, but window length becomes a key trade-off; short windows react faster but are noisier, while long windows are more stable but blur transitions and may mix different activities. Third, lag-aware alignment: because EDA and cardiovascular responses can peak at different times, some pipelines include time-shifted versions of one modality so the model can learn whether the informative offset is at *t*, t+Δ, or t−Δ. Finally, early fusion must also define how to handle missing data (e.g., brief sensor dropouts), since concatenation is ambiguous when one stream is absent.

Zhang et al. directly targeted this issue by framing fusion as an alignment problem rather than a concatenation challenge. Their model processes BVP and EDA with modality-specific feature extractors (1D-CNN for quasi-periodic BVP and Long Short-Term Memory (LSTM) network for the much slower EDA signal), then uses bidirectional cross-modal attention to learn where to align time slices from one modality to the other. Instead of enforcing alignment by resampling, the model learns alignment implicitly by assigning attention weights over time (to be addressed in Section 5), filtering the noise and redundancy induced by fusion, as shown in Figures 2 and 8 in reference [59].

### 4.2. Feature-Level (Intermediate) Fusion: The Dominant Paradigm, Decoupling Sensing from Learning

Feature-level fusion is the default workflow in multimodal stress detection due to its ability to avoid the issues previously defined by raw-signal fusion while still preserving the useful information needed. Each modality is first converted into compact sets of features over a time window, and then those feature blocks are concatenated into one vector for classification. This is the standard workflow behind “multimodal feature analysis” in applied settings, where models operate on engineered descriptors rather than raw signals [24].

In practice, feature-level fusion is also where the main failure modes emerge:

- Dimensionality increases.
- Feature relevance becomes person dependent.
- Easy overfitting on machine learning models trained on limited data.

Kalinkov et al. describe this directly: researchers build composite feature vectors because no single feature is sufficient, but dimensionality can degrade robustness, which is why feature selection, rather than optimization, becomes a priority. Their adaptive Fisher discriminant ratio (FDR) method is a clean example of filter-based selection targeting improved classification while reducing the effective feature set [26]. Across the literature, fused feature vectors draw from a recurring set of families:

- EDA features (tonic and phasic): Skin conductance level (SCL) baselines/means, slope and variance, and phasic SCR event-like descriptors (number of peaks, amplitude, rise/fall dynamics). Often used as the “anchor” features due to directly tracking to sympathetic sweat gland activity.
- Cardiac features (ECG/PPG HRV): Heart rate and variability metrics in time and frequency domains (e.g., IBI, HR bandpower ratios [low-frequency/high-frequency]). Kalinkov’s examples include HRV as a power ratio feature, paired with skin resistance/EDA features in multimodal sets [26].
- Respiration/wave morphology: Respiration rate and variability and PPG pulse wave descriptors are commonly added when additional context is needed if EDA + HR information saturates.
- Frequency domain descriptors: Some papers move beyond “features per modality” and treat frequency structure as a shared space. Radhika et al. extract features from power spectral density (PSD) across ECG and phasic EDA bands, then the model learns joint representations from the concatenated feature sets, reporting a subject-independent focus [16].

Beyond just model performance, feature selection also plays a role in determining whether the model is ready for wearable deployment:

- Cost-awareness feature selection: Momeni et al. frame multimodal stress monitoring as a trade-off between accuracy and battery life, primarily due to the high cost of multimodal data acquisition/processing. Their CAFS formulation selects features under energy budgets (including cost dependencies) and shows that simple single-signal rules (exclusively HR or SCL) can be confidently used in narrow regimes, but outside those regimes a multimodal feature set (including respiration and pulse-wave features) is required for confidence in classification [38].
- Feature relevance and dependence: Li et al. argue that conventional selection methods treat stress-like states as discrete bins, but in reality stress is continuously evolving. They propose selecting features based on transitions between states (before vs. after a transition) and report that their method reduces the feature set to 13 total features across ECG, PPG, and GSR while still improving recognition compared to baseline approaches [42].

Feature-level fusion partially sidesteps the synchronization problem stated earlier by moving fusion from the sample level to the window level. Instead of forcing raw streams to line up point-by-point, each modality is summarized over the same time interval (e.g., mean/variance, peak statistics, spectral power, or learned window embeddings), and these window features are then concatenated for classification. This makes disparate sampling rates less problematic, but it makes window length a primary design choice. Short windows (e.g., 30 s) improve responsiveness and can capture brief stress transients, but they are more sensitive to motion artifacts and may not contain enough cycles for stable cardiovascular estimates. Moderate windows (e.g., 60 s) often provide a better balance for multimodal fusion, reducing noise while still tracking changes over time. Longer windows (e.g., 5 min) stabilize slower descriptors, especially HRV features that require sufficient inter-beat data, but they blur temporal boundaries, increase label ambiguity when stressors are brief, and can mix physiological markers across different activities. Across the reviewed studies, window sizes are typically selected to match the slowest modality being used (most commonly HRV), the expected duration of the stress induction or annotation interval, and the desired update rate of the system. As a result, reported gains from feature-level fusion should be interpreted in light of the windowing protocol, since the same fusion strategy can behave very differently under 30 s versus 5 min aggregation.

In all, feature-level fusion dominates because it is implementable, flexible across sensor suites, and compatible with both machine learning and modern deep learning methods (discussed in Section 5). The field is quickly converging on the same conclusion: once sensors are fused, feature selection determines the performance of a model. It determines generalization, battery life, and the system’s ability to survive outside a curated lab space [26].

### 4.3. Decision-Level (Late) Fusion: Ensemble Methods and Voting Schemes for Reliability

Decision-level (late) fusion mitigates the risk of a single noisy sensor compromising the entire system by employing ensemble techniques that treat each modality as an independent expert, aggregating their predictions to form a final consensus. In wrist wearables, at least one channel has a chance of being wrong at any given time, whether due to bad contact, motion, missing data, or users who do not express arousal strongly in that modality. Late fusion limits these risks.

Mozafari et al. make the argument explicitly in a cross-subject setting. They point out that standard feature concatenation plus feature reduction can overweight one modality and make a system brittle when that modality is providing low-quality or uninformative data. Their proposed solution is late fusion. They trained modality-specific classifiers, then merged them into a score-level model that combined scores rather than giving hard labels [57]. In their experiments, this fusion strategy is framed explicitly for robustness to inter-subject variability and modality-specific noise. By combining class confidence scores rather than hard labels, the model reduces dependence on any single signal stream and dynamically downweights modalities that are noisy or uninformative in a given trial. They report that this score-level fusion improves five-class stress classification performance relative to feature concatenation baselines [57].

Simic et al. provide another practical example of late fusion in daily-life wrist pipelines, where physiological inference is fused with self-reported information at the output [61]. Their architecture separated the passive physiological prediction (from GSR, HRV, SKT, and SpO_2_) from active user-reported signals and then combined them using a late-fusion rule (weighted combination) to better align the output with perceived stress [61]. The key idea remains to keep streams separated until the very end and then combined them in a way that is robust to disagreement between modalities. In real-world deployment, this creates an interpretable failure mode. When physiology and self-report diverge, it is noticeable rather than hidden inside a single fused feature vector.

**Figure 6:**
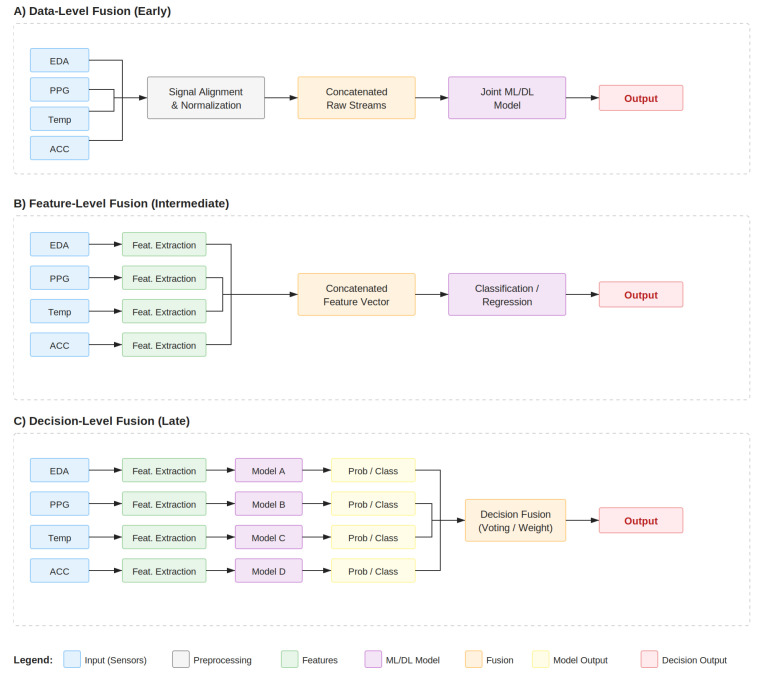
Example comparison of multimodal fusion strategies for wearable stress inference. Early fusion combines raw signals prior to modeling, feature-level fusion concatenates modality-specific features before classification, and decision-level fusion aggregates modality-specific model outputs via voting or weighted rules.

Late fusion is used when reliability matters more than marginal accuracy (see Figure 6). It is the most natural fusion strategy for noisy wearables because of it achieves the following:

1. Isolates sensor-specific failure
2. Supports dynamic reweighting based on signal quality
3. Avoids domination of one modality

### 4.4. Comparison of Strategies: When to Use Which?

There is no universal consensus on the “best” fusion strategy. While feature-level fusion currently offers the optimal balance of performance and complexity, comparative studies indicate that the choice of architecture is highly influenced by the specific application context and the available computational resources. The choice boils down to a systems decision based on what signals you have, noise levels of signals, time alignment, and whether evaluation is done on-device or is processed offline.

Data-level (early) fusion is most appropriate when synchronization is trivial and when the model is intended to learn interactions between raw signals. The simplicity comes at the cost of increased fragility when raw signals have different sampling rates or noise profiles. Early fusion can quietly fail for alignment or signal quality. In comparative experiments, early fusion tends to train faster rather than heavier ensemble setups, but can quickly underperform compared to the best late-fusion models [17].

Feature-level (intermediate) fusion is the default when the goal is strong performance with manageable complexity. Decoupling sensing from machine-learning algorithms, each modality is cleaned and summarized, then windowed for representation. At that point, the signals are concatenated into a single feature vector. This is practical when the signals are varied (EDA vs. ECG vs. SKT) and when computing power is limited, which is common on wrist wearables. Feature extraction can be designed to be lightweight and robust, and feature-level fusion can also be easy to debug and interpret. When performance drops, it can still be traced back to singular sensors, feature families, or a preprocessing step. A common theme in the wearables industry is the computational constraints that shape what is feasible. Rodrigues et al. combated this by reducing volume via windowing because raw streams are typically too large to handle directly in a machine learning loop, and they showed that more costly processing choices can cost time without clear measureable gains [28].

Decision-level (late) fusion is found to be most useful when one cares about reliability under failure or noise. In this case, each modality produces its own prediction, and the system aggregates them (often weighted). This “sensor independence” framing is robust. If one sensor is corrupted, it does not put the entire system at risk. In Eldien et al., late fusion produced the best overall performance (by F1-Score), outperforming intermediate (feature/model level) fusion by 0.0197 and early (data level) fusion by 0.0126, but it also required training multiple models and carried a larger footprint, which is costly for low-power wearables [17].

Quantitative comparisons across the reviewed studies reinforce these qualitative distinctions while revealing that the magnitude of fusion benefit varies substantially by architecture. At the decision level, Eldien et al. directly compared all three strategies on a real-world nurse stress dataset and reported that prediction-level (late) fusion achieved an F1 of 0.6716, outperforming model-level fusion by 1.97 percentage points and data-level (early) fusion by 1.26 percentage points [17]. Although the gains are modest, the direction is consistent with the reliability argument: late fusion was less susceptible to the noise inherent in hospital shift data. In contrast, Radhika et al. demonstrated that joint-modality fusion via an autoencoder-based shared subspace consistently outperformed unimodal baselines and both early and late fusion strategies in subject-independent evaluations across four datasets, with absolute accuracy and F1-score improvements ranging from approximately 0.08 to over 0.20 depending on dataset and comparison baseline. These gains were most pronounced relative to unimodal and traditional feature-based pipelines, indicating that learned joint representations capture cross-modal structure that output-level aggregation alone fails to exploit [16].

In practice, if one prioritizes deployability and cost-aware pipelines, feature-level fusion is a good start, with no need to escalate unless preprocessing issues cannot be fixed. If an application is safety-critical or sensors may drop out, late fusion often addresses these complexities. If prototyping and learning are still early, feature-level fusion can offer a clean path to ablations and can quickly expose cases where signals are weaker than expected. Rodrigues et al., for example, reported low performance when relying on EDA, while ECG/EMG combinations performed better [28].

## 5. Algorithmic Inference Models

### 5.1. Statistical and Rule-Based Approaches: Thresholding and Simple Correlations

Before the adoption of complex deep learning, SNS arousal detection relied on statistical analysis and explicit rule-based logic to interpret physiological data. The fundamental challenge in this domain is establishing a reliable “ground truth,” as physiological responses are highly idiosyncratic; a baseline heart rate for one individual may represent an arousal state for another. Consequently, early approaches focused on transforming raw, noisy biosignals into standardized metrics, known as “features”, that could be objectively compared across different people and situations [52].

The primary task in explicit feature extraction is “feature engineering,” where specific characteristics are mathematically extracted from raw signals to summarize the body’s state:

- Time-Domain Features: These are statistical summaries calculated directly from the signal waveform over a set period. Common metrics include mean, median, maximum, variance, and standard deviation. For example, in galvanic skin response (GSR) and photoplethysmography (PPG) [31], an increase in standard deviation often indicates the higher variability associated with physiological arousal.
- Frequency Domain Features: These metrics analyze the rhythm of the signal rather than just its amplitude. This is particularly critical for heart rate variability (HRV), where the signal is decomposed into low-frequency (LF) and high-frequency (HF) bands. These bands serve as proxies for the autonomic nervous system: LF is often associated with the sympathetic (“fight or flight”) system, while HF reflects parasympathetic (“rest and digest”) activity [35,49].

To ensure these features are accurate, “rule-based” pre-processing and filtering is essential for cleaning the data before analysis. Biosignals are notoriously noisy; movement or loose sensors can create artifacts that mimic arousal responses:

- Artifact Removal: Methods such as interquartile range (IQR) are applied as a data filtering process to eliminate outliers and artifacts from noisy signals. By identifying and removing data points that fall statistically far outside the “normal” range (outliers), these rules preserve the core biological signal while discarding noise caused by motion or hardware errors [49].
- Peak Detection: Accurate feature extraction often depends on identifying specific points along a signal, such as the peaks in a PPG wave or skin conductance responses (SCR) in EDA. Rule-based algorithms define specific criteria (e.g., amplitude threshold and minimum distance between peaks) to reliably count these events, which are the prerequisites for calculating HRV and pulse rates [35].

To address the issue of human diversity, normalization techniques are applied to make absolute values comparable across different individuals. A widely adopted method is Z-standardization (z-norm). This process rescales the feature vectors by centering them around an individual’s specific mean and dividing by their standard deviation. By eliminating the absolute mean value and unifying the dynamic range, z-normalization allows the model to detect relative deviations from a person’s baseline, identifying when a user’s arousal state is “elevated relative to themselves,” rather than relying on arbitrary universal thresholds. This process was shown to be impactful by Romine et al. [29]. Normalization enables robust comparison by compensating for differences in baseline physiological responses [52].

### 5.2. Classical Machine Learning: The Efficiency of SVM and Random Forests in Wearable/Battery- Constrained Devices

Despite the rapid emergence of deep learning, classical machine learning (ML) algorithms, specifically Support Vector Machines (SVM) and Random Forests, remain the dominant paradigm for battery-constrained wearable devices. Unlike deep neural networks, which function as “black boxes” requiring immense computational power, classical algorithms offer a predictable computational footprint and inherent interpretability [32,50], making them ideal for the limited hardware of smartwatches and fitness trackers. These conventional methods are uniquely advantageous for on-device processing, where managing energy consumption is as critical as accuracy.

The application of fundamental classifiers demonstrates that complex architectures are not always necessary to handle multimodal biosignals effectively. Simple algorithms like K-Nearest Neighbors (KNN) classify data by finding the “k” closest data points (neighbors) to a new point and using their labels/values to predict the new point’s outcome. For instance, a recent study used these algorithms to analyze multimodal signals comprising electrodermal activity (EDA) and skin temperature (SKT), achieving an F1-score of up to 0.8 and classification accuracy exceeding 0.70 [47]. The mathematical simplicity of these traditional algorithms makes them highly scalable and reliable for continuous, real-time monitoring, allowing devices to output rapid predictive outcomes without draining their batteries [43].

The success of classical ML relies on upstream signal processing and explicit feature engineering to transform raw signals in discriminative metrics [50]. While deep learning automates this process, classical ML requires researchers to identify the most impactful inputs manually.

- Feature Selection: Techniques such as recursive feature elimination (RFE) are employed to mathematically rank and select the most significant parameters from complex signals [31].
- Advanced Transformations: Other studies leverage sophisticated mathematical transforms, such as the General Linear Chirplet Transform (GLCT), to extract time-frequency features from EDA and PPG signals [40].

These analyses (spanning time, frequency, and nonlinear domains) generate a compact “feature vector”, a summarized numerical fingerprint of the signal, that accurately captures the correlations between physiological changes and stress states.

This methodology focuses on mathematical refinement of input. For example, studies employ techniques such as recursive feature elimination (RFE) to identify the most discriminative features for stress classification from ECG, GSR, EDA, and PPG signals [31], while others leverage the General Linear Chirplet Transform (GLCT) to extract time-frequency features from EDA and PPG prior to classification [40]. By decomposing signals using GLCT and subsequently extracting robust statistical parameters, researchers achieved high classification accuracy (0.9213 using RF) in differentiating cognitive states [40]. This feature set, often derived from multi-domain analyses (time, frequency, and nonlinear domains), gives way to the final compact feature vector that accurately captures the correlations between physiological changes and stress states [53].

When optimized and paired with high-quality features, these non-deep learning approaches yield predictive metrics that compete with, or even outperform, deep learning architectures in subject-dependent or resource-constrained environments [31,43]. For example, the use of physiological and psychological features as inputs for a bootstrapping algorithm, an ensemble method that aggregates multiple models to improve stability, achieved highly compelling results in binary stress classification: an accuracy of 0.9582, precision of 0.9933, and an F1-score of 0.9469 using a subject-dependent random train/test split on the WESAD dataset, a setting that allows subject overlap between training and evaluation sets [43]. This highlights the continued relevance of classical ML in scenarios where computational efficiency and interpretability are prioritized.

### 5.3. Deep Learning Advances

Convolutional Neural Networks (CNNs) have transformed the feature engineering pipeline by enabling the automatic extraction of spatial patterns and high-level representations directly from raw biosignal spectrograms, facilitating sophisticated pattern recognition without demanding manually crafted features [41]. This approach contrasts sharply with traditional pipelines by leveraging deep network architectures to learn data representations autonomously from various inputs.

#### 5.3.1. CNNs for Feature Extraction

Stress detection has long relied on “feature engineering”, where researchers manually calculate metrics, such as the mean amplitude of a skin conductance response or heart rate variability, as these serve as inputs for classification models. While effective, these methods can easily overlook hidden non-linear information within the physiological signals. To address this, recent work has leveraged Convolutional Neural Networks (CNNs). Originally created for image processing, CNNs are increasingly used in physiology to automatically learn patterns from raw sensor data. This process uses automated feature discovery, acting as a mechanism to extract hierarchical patterns directly from physiological data. By sliding filters over the input, CNNs can exploit the invariance of stress markers, identifying anomalies regardless of their specific location within the observation window.

While originally designed for computer vision, CNNs are applied to stress detection by treating physiological signals as analogous to images. This is achieved through two primary approaches: 1D Convolutions, which slide filters directly over raw time-series data to detect local anomalies like QRS complexes or SCR peaks; and 2D Convolutions, which process transformed representations such as spectrograms or scalograms. By converting the signal into a time-frequency image, the CNN can utilize its translation invariance to identify stress-related spectral patterns regardless of their specific location in the window.

Meneses et al. validated the approach of 1D CNNs for stress detection using raw signals, directly applying kernels to detect local features of a temporal signal. Using a CNN with three convolutional layers to extract maps from EDA and SKT, their model used global average pooling (GAP) to compress these convolutional features into a dense representation, achieving 0.99 accuracy on the WESAD dataset. This highlights the CNN’s ability to automate feature selection without extensive preprocessing [36].

Lee et al. (2024) provided one of the clearest quantitative demonstrations of multimodal advantage within an attention-augmented deep neural network framework [19]. Using a residual attention-based deep neural network (DNN), evaluated under leave-one-subject-out (LOSO) validation on the WESAD dataset, they reported that fusing all available modalities (EDA, PPG, skin temperature, respiration, and accelerometry) achieved 0.9657 accuracy, compared to 0.9289 for EDA alone and 0.9216 for PPG alone, corresponding to gains of 3.68 and 4.41 percentage points, respectively [19]. The attention mechanisms within the network integrate raw biosignals with human-engineered features and learn data-dependent channel and temporal weighting, enabling the model to emphasize the most informative inputs for each window. This result is notable not only for the absolute performance improvement, but also for demonstrating that even when unimodal EDA performance already exceeds 0.92, multimodal fusion combined with attention mechanisms can extract additional discriminative information that is not fully captured by any single sensing channel.

**Figure 7:**
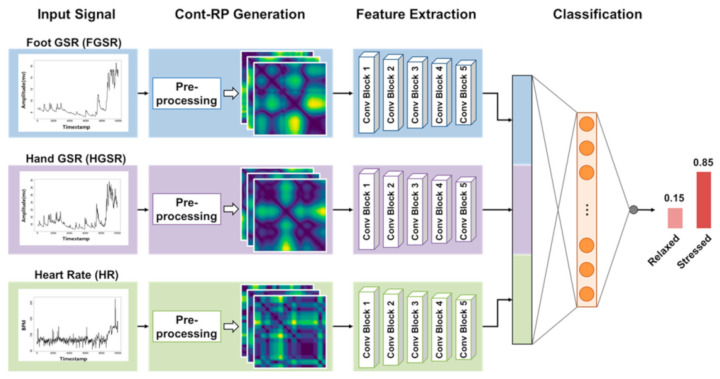
Continuous recurrence plot (Cont-RP)-based deep learning pipeline for multimodal stress inference. Raw physiological signals (foot GSR, hand GSR, and heart rate) are preprocessed and transformed into continuous recurrence plots, which encode nonlinear temporal dynamics as spatial textures. Modality-specific convolutional feature extractors learn hierarchical representations from each Cont-RP prior to joint classification. Reproduced from [34]. Licensed under CC BY 4.0.

Expanding beyond 1D signals, Lee et al. (2021) proposed converting time-series data into 2D images called continuous recurrence plots (Cont-RPs), as shown in Figure 7 [34]. These plots visualize the recurrence of physiological states over time, revealing non-linear textures such as “netting” or “bubble” patterns that appear during high-stress scenarios, while relaxed states appear as smooth and monotonous transitions. Feeding these Cont-RPs, which consist of heart rate and GSR data, into CNNs enabled the model to detect stress with a reported accuracy of 0.9567, even with small signal windows of 30 s or less. This demonstrates the robustness of CNNs in capturing complex temporal dynamics through spatial representations [34]. Meneses et al. report classification accuracies of up to 0.99 on the WESAD benchmark and 0.87–0.90 on pilot simulator data, though these results reflect subject-dependent evaluations in which training and test data are drawn from the same individual [36]. By contrast, Lee et al. evaluated their attention-based model under leave-one-subject-out cross-validation, achieving accuracies of approximately 0.95–0.96 despite complete subject separation, indicating stronger generalizability [34].

The effectiveness of CNNs relies heavily on the quality of the features and the depth at which they are integrated. Radhika et al. investigated this by comparing early, intermediate, and late fusion for EDA and ECG signals. Their findings suggest that intermediate fusion, concentrating features after the fourth convolutional layer, yields the highest performance (0.85 accuracy), indicating that CNNs learn much more abstract and discriminative features in deeper layers, which are more effective for stress classification than raw or shallow features [62].

Recently, researchers have explored integrating attention mechanisms within CNNs to refine feature extraction. Lee et al. (2024) [19] proposed a Residual Attention Network that combines raw biosignals with human-engineered features, such as EDA peaks, PPG rate, ACC, and SKT. The attention modules within the CNN learn to weigh these features dynamically, focusing on the most relevant patterns for stress detection. This approach achieved an accuracy of 0.9657 on the WESAD dataset, demonstrating how attention can enhance CNN feature extraction by emphasizing critical physiological markers.

#### 5.3.2. LSTMs/RNNs for Temporal Dependencies

Recognizing that stress is a dynamic, time-varying phenomenon, researchers have increasingly utilized Long Short-Term Memory (LSTM) networks to capture the temporal dependencies and long-term trends inherent in physiological data. Unlike models that treat inputs as snapshots independent of each other, Recurrent Neural Networks (RNNs) and LSTMs address the sequential nature of SNS arousal and can model how stress responses evolve over time.

The novelty of LSTMs lies in their gating mechanisms, which allow for the network to retain relevant information while disregarding noise. This is essential in order to accurately model physiological changes over time, capturing the gradual fall of SCR peaks following a stressor.

To capitalize on this, Meenalakshmi et al. proposed a multigate LSTM as an advanced variant designed to regulate information flow with higher precision. By processing multimodal inputs such as EDA, PPG, HR, and accelerometer data, the model was shown to adapt to complex non-linearities of physiological signals, achieving a Root Mean Square Error (RMSE) of 0.286 in stress estimation [54].

Another study by Halder et al. employed a more standard approach, as LSTMs work well in direct sequence classification tasks. The group utilized the LSTM approach on the Stress-Predict dataset, which includes EDA, HR, ACC, and IBI. The model showed the ability to differentiate between stress and non-stress states with an accuracy of 0.9178 [46].

Both of these studies took advantage of the LSTM’s ability to model temporal dependencies and their ability for generalization. Abdelfattah et al. [50] highlighted that while conventional neural networks often struggle in cross-subject scenarios (such as seen by the groups Phase 1 studies), deep recurrent architectures greatly outperform them, as they capture universal stress patterns better, leading to more robustness across different individuals. The scale of cross-subject performance degradation and the computational cost of mitigating it are both quantifiable. Abdelfattah et al. illustrated the former by reporting an F1-score of 0.998 on WESAD using an XGBoost classifier under an 80:20 train/test split, a result achieved with a classical tree-based model rather than a deep recurrent architecture, with performance reductions on the order of several percentage points depending on model architecture and evaluation phase [50]. In contrast, recurrent neural networks required 564 s of training time, compared to 0.13 s for Gaussian Naïve Bayes, a disparity of more than three orders of magnitude, without yielding superior performance in the subject-overlapping setting. This highlights a recurring tension: while deep temporal models can capture richer sequential dynamics, their computational overhead does not necessarily translate to proportional gains, particularly on small datasets with subject overlap. Nkurikiyeyezu et al. quantified the generalization failure more directly by evaluating models on four completely unseen subjects. Their HRV-based workload prediction model achieved an out-of-sample Mean Absolute Error (MAE) of 10.37 with no adaptation, a level of error that limits its utility for individual-level inference. However, incorporating just 25 calibration samples per new user reduced MAE to 6.31, and 125 samples further reduced it to 2.81, with classification precision increasing from 0.3583 to 0.9373 [13]. Together, these results define the practical cost of subject independence: either models accept substantial degradation on new users, or systems must incorporate a brief calibration phase to recover reliable performance.

#### 5.3.3. End-to-End Hybrid Architectures

The ongoing refinement of stress detection methods has led to the adoption of sophisticated “end-to-end” deep learning models. These architectures are designed to handle the complex, time-varying nature of physiological signals by processing raw data streams directly, rather than relying on manually isolated features. By integrating different types of neural networks, such as those that recognize patterns (CNNs) and those that track changes over time (Recurrent Networks and Transformers), researchers can maximize classification accuracy and capture the full dynamic picture of human stress.

Hybrid Convolutional and Recurrent Architectures have become a predominant approach, which involves fusing CNNs with LSTM models. This was done by Naga Pawan et al. [60] to address two simultaneous requirements: extracting detailed local features from short signal segments and modeling how these features evolve over time.

- Synergistic Feature Learning: The CNN layers in this architecture function as feature extractors, identifying spatial patterns (such as specific waveform shapes in ECG or EDA), while the LSTM components analyze the temporal sequence of the signals. This effectively allows for the merging of spatial detail with long-term context retention.
- Performance: The hybrid model consistently outperformed standalone architectures. For instance, the CNN–LSTM hybrid model applied to the WESAD dataset achieved 0.96 accuracy (compared to a max of 0.94 on a standalone BiLSTM model), a clear indicator that integrating spatial and temporal signals provides a more robust framework for stress detection.

To further enhance accuracy, research has pivoted to “Attention” and Transformer based architectures. Transformers utilize self-attention to weigh the importance of different time steps globally, allowing for the model to focus on the most relevant segments of the input sequence when making predictions. This is particularly advantageous for stress detection, where certain physiological events (like sudden SCR spikes) may carry more significance than others.

Transformer–CNN fusion as proposed by Li et al. [41] represents an approach that combines the strengths of both architectures. The CNN layers extract local features from raw signals, while the Transformer layers apply self-attention mechanisms to capture long-range dependencies across the entire sequence. This dual approach allows for a comprehensive analysis of physiological data. Tested on pilot stress data, this method achieved a binary classification accuracy of 0.9328, confirming that attention-based modeling can effectively handle the complex temporal nature of multimodal physiological data.

Modality Awareness and Alignment: Handling data from different sensors (such as heart-rate and EDA) often introduces challenges related to noise and temporal misalignment. Abe and Jung [65] addressed this by developing “Modality-Aware” Transformer models that incorporate specialized attention mechanisms to align and weigh inputs from different modalities effectively. This approach ensures that the model can focus on the most informative signals while mitigating the impact of noisy or irrelevant data. Similarly, Zhang et al. [59] developed a Dynamic Alignment and Fusion framework utilizing Bidirectional Cross- and Self-Modal Attention (BCSA). This mechanism actively aligns temporally mismatched signals and filters out redundancy, producing a unified and salient representation of stress.

Developing modalities such as Spiking Neural Networks (SNNs) have emerged as a powerful potential alternative to traditional deep learning. SNNs mimic the discrete, spike-based information processing of the human brain, offering high energy efficiency and low latency. B S, Rao, and Pavan K [20] proposed a neuromorphic stress detection system utilizing graph-based SNNs, achieving a remarkable accuracy of 0.9949 on the WESAD dataset while significantly reducing energy consumption compared to standard neural networks. To further refine this approach, B S et al. [21] introduced the Quantized Deep Evolutionary SNN (MC-QDSNN), which incorporates Multicompartment Leaky (MCLeaky) neurons. These biologically inspired neurons allow for non-linear processing within the spiking domain, enabling the model to capture complex temporal features with lower computational overhead than traditional Spiking LSTMs.

## 6. Context-Awareness and Robustness

### 6.1. Motion Artifact Handling: How Multimodal Systems Distinguish “Running” from “Stress”

Differentiating between physiological arousal driven by physical exertion and that caused by acute psychological stress remains a primary challenge in ambulatory settings, necessitating robust motion artifact handling. In daily life, high arousal is not always the result of stress, but can also come in the forms of excitement, physical exertion, or intense emotional states like fear or anger. EDA can shift due to heat and changes in skin contact. If models only learn about HR and EDA levels to classify stress, the models can overfire on classification and can miss true stress when movement occurs.

Robust multimodal systems must then use movement as context for classification. Askari et al. framed this by detecting both unannounced physical activity and acute psychological stress events using a wrist sensor stack that included accelerometry (ACC) and physiological changes (PPG + EDA) [37]. The important idea is not just adding ACC as another feature but using it instead to explain physiology. If evidence of excessive movement is given, a high heart rate should be expected and is not interpreted the same way a stressor at rest is. This helps multimodal systems avoid confusion of exercise for stress.

Even when the user is doing the same activity, real-world motion artifacts often arise from the sensor itself. Shifts of the sensor during movement can change signal distribution enough to change state evaluation that during controlled studies was deemed strong. Simons et al. quantified this “sensor variance” problem and showed that changes in sensor type and placement can degrade the accuracy of a model, even when modeling the same events [30]. This matters because it frames artifact handling as a matter of designing wearable devices that can survive the variance of sensors during daily use.

Mobility introduces noise sources, motion artifacts, environmental variability, and rapid transitions between contexts (walking, stopping, turning, etc.). Kalimeri and Saitis addressed stress detection during indoor mobility and showed why multimodal feature sets are needed when the user is actively moving [23]. Their work supports a practical takeaway that robust ambulatory stress inference requires the following:

- Explicit movement awareness.
- Fusion strategies that reduce reliance on any one channel.

The quantitative evidence for context-aware motion handling supports this recommendation. Gjoreski et al. demonstrated that incorporating activity-level context into a multimodal stress detection pipeline substantially reduced false positives in real-life, event-based evaluation. The number of true negatives increased from 638 to 790, indicating that many instances of physiological arousal previously misclassified as stress were correctly attributed to physical activity when contextual gating was applied [4]. Notably, this improvement required no additional sensors, but rather a reinterpretation of existing accelerometry data as contextual input to the stress classifier. Rahman et al. demonstrated a complementary physiological disambiguation strategy using earbud-based sensing: while heart rate and HRV features alone achieved an F1-score of 0.8512, augmenting the model with respiration rate and core body temperature, signals sensitive to exertion-induced autonomic changes improved F1 to 0.9078 under leave-one-subject-out evaluation, a gain of 5.66 percentage points [39]. At the deployment level, Gibbs et al. showed that such context-aware gating is feasible even on severely resource-constrained hardware, achieving 0.88 stress detection accuracy on an Arduino Nano 33 Sense by running separate TinyML models for activity recognition and stress classification, with the activity model determining when physiological inference should be invoked [58]. Together, these results indicate that motion artifact handling is not merely a preprocessing concern, but a system-level design decision that measurably improves both classification reliability and real-world deployability.

### 6.2. Contextual Adaptation: Using Location/Time to Adjust Thresholds (e.g., “Office” Mode vs. “Home” Mode)

To move beyond laboratory constraints, recent research has focused on contextual adaptation, employing methods that adjust inference models based on the user’s environmental surroundings, such as the transition from a home to an office setting. Models no longer assume a fixed environment, and in doing so, the system treats context as part of the input to the inference problem.

Can et al. provided one of the clearest examples of this in practice. They deployed smart bands in a real-life multi-day setting and showed why lab-trained pipelines degrade. Their contribution is explicit in that combining physiological features (EDA, HRV, SKT, etc.) with context variables (physical activity level, activity type, weather, etc.) shows that context improves stress inference across session-based and longer-horizon labels [27]. They also provided concrete architecture that a wearable engineer can implement.

Upon accepting that context changes the meaning of physiology, two major engineering strategies emerge:

- Model switching.
- Domain adaptation.

Gibbs et al. demonstrated model switching in a binary deployment sense. Rather than running one larger model, they ran multiple small models on-device (TinyML). Their approach was direct in that they first inferred physical activity continuously to identify periods where PPG/EDA were likely to be corrupt, then gated or conditioned stress inference accordingly [58]. The sense of context awareness is a decision variable that determines whether stress inference should run and which model is more likely to be accurate. It also matters as it pushes context awareness into resource-constrained wearables, where you cannot afford heavy models or constant cloud inference [58].

Mihirette et al. formalized domain adaptation across-context prediction. By training ML models in one domain (such as a lab setting or dataset) and then using the model in another domain (driving, nursing, etc.), their framework lays out a methodology for this transition: (1) choose shared vital signs across domains (HR, HRV, EDA, or TEMP); (2) extract the features, (3) identify significant predictors, then (4) apply domain adaptation techniques such as feature-space alignment (CORAL in combination with Random Forest) to reduce distribution mismatch [55]. The shift in context mathematically is a shift in distribution, so if feature distributions move, then the model must adapt to the new setting or it will fail.

Context awareness appears to be maturing quickly in the domain of automotive driving. Driving can be high-stakes, and the environment is well-structured enough such that stressors can be defined, with safety as the primary argument. Singh et al. explicitly framed real-time stress-trend detection as a wearable computing problem, constrained by computation cost, battery, and memory, and emphasized the need for online (real-time) detection of stress changes during driving scenarios using GSR, PPG, and engineered features [22]. Bianco et al. then reflected on a more modern multimodal version of this pipeline. Multiple physiological channels (HR, BR, EDA, etc.) are windowed, features are extracted, and stress is inferred under both cross-validation and leave-one-subject-out settings, with multimodal feature concatenation and stacking used to improve performance [25]. Even if modeling choices vary across studies, the domain-level message remains that context-aware stress inference is less about demonstrating that physiological arousal correlates with stress labels, and more about building systems that can reliably distinguish stress-related arousal from baseline or task-specific conditions under movement, device variability, and operational constraints [22,24,25]. A second mature high-stakes domain is within the healthcare sector, where context is dynamic (shift work, physical movement, and repeat acute stressors) and the cost of failure is high. Mathur et al. provided a concrete example in nursing using multimodal wearable signals (PPG and EDA) and comparing single-modality models to multimodal models, motivated by the need to monitor stress in hospital environments [27,48].

The deployment of context-aware sensing has reached a mature stage in high-stakes environments, particularly within automotive and occupational healthcare sectors, where real-time stress inference is critical for safety.

### 6.3. Subject-Independence: The Challenge of Generalizing Models to New Users

Achieving subject independence is a critical hurdle for scalable technology, as physiological baselines vary significantly between individuals, often requiring models that can eliminate subject-specific bias to generalize effectively. A subject-dependent model can look “solved” because it learns the specific user, your typical response and recovery habits. However, once this model is deployed on a new user, the implicit assumptions are no longer valid.

Nkurikiyeyezu et al. make this explicit with a test often overlooked, evaluating unseen users. Their models were trained on EDA or HRV and achieved extremely strong cross-validation performance on known users, but performance was hindered when tested on new subjects. This shows that physiological fingerprints differ enough across people that generic stress models exhibit high generalization error without adaptation [13].

This does not mean that subject-independent stress detection is not feasible, but does mean that subject independence is not a single model choice. Nkurikiyeyezu et al. showed that adding even a small amount of data from unseen users (bootstrapping “seed samples”) can significantly improve performance, which supports the idea that real systems need a certain amount of personalization, calibration, or adaptation [13].

Das et al. [18] approached the same problem from a different angle as they removed individual bias by reorganizing the training population. They showed that early fusion of multimodal features (EEG, PPG, and GSR) improves leave-one-subject-out performance relative to EEG alone, but they also went further by clustering subjects using self-reported valence arousal dominance (VAD) patterns. In their results, this bias-aware clustering improved LOSO F-scores beyond just fusion and also suggested a practical strategy of, instead of forcing one universal mapping, learning mappings of cohorts of similar responders.

Sah et al. highlighted why subject independence becomes more difficult in real-life settings. Daily, as more users are introduced, so are unknown variables such as stress timing, delayed self-report markers, inconsistent context, and shifting sensor quality. They tackled this by modeling the time uncertainty between user-marked stress events and the actual physiological peak, then showed that design choices like window length and class balancing materially change measured performance. They also showed why systems that rely on sensors are expensive and difficult to maintain at scale, pushing the field toward selecting the smallest sensor set that still generalizes [44].

Taken together, these papers define subject independence as a generalization problem with three concrete sources of failure:

- Baseline and response variability across individuals;
- Label subjectivity and reporting bias;
- Uncontrolled free-living noise and timing uncertainty.

The implication that “universal stress models” will not work reliably unless they include an adaptation mechanism is consistent. Whether a short calibration phase is required, cohort-aware modeling adaptation, or continuous personalization that learns a new user’s physiological fingerprint over time, is required [13,18,44].

## 7. Discussion and Future Directions

### 7.1. The “Battery vs. Accuracy” Trade-Off

The emergence of deep learning (DL) in modern technology has undeniably transformed the way researchers can create benchmarks for stress classification, yet this potential comes at the significant cost of computational energy [19,64]. While complex models like Transformers and Residual Attention Networks can achieve high accuracies (0.96), high memory footprints often exceed the capabilities of ultra-low-power microcontrollers [19,58]. Researchers are increasingly forced to choose between “Full” modes that utilize a wide array of signals for maximum confidence and “Budget” modes that prioritize battery longevity by selecting only the more energy-efficient features. Momeni et al. quantified this by reporting that a full multimodal model (ECG, RSP, PPG, and EDA) consumed 85.0 mJ per 60 s inference window, whereas an optimized “budget” model using only essential features reduced consumption to 8.75 mJ, an energy saving of approximately 90% but at the price of a drop in prediction accuracy [38]. B S et al. demonstrated that neuromorphic Spiking Neural Networks (SNNs) on the Intel Loihi-2 chip achieved energy savings of 25.12× to 39.20× compared to equivalent Artificial Neural Networks (ANNs) running on an Nvidia Jetson Nano edge GPU. Integration SNNs and quantized models represent a good middle ground, potentially reducing computational cost, in Floating-point Operations per Second (FLOPS), by 2.4× compared to traditional DL networks [21]. Ultimately, for a system to be ready for long-term use, the field must pivot toward self-aware monitoring that triggers high-power multimodal classification when baseline “budget” models lack confidence [38]. To further conserve resources, Simic et al. implemented duty cycling, activating power-hungry PPG and GSR sensors for only 60 s every 5 min when at rest [61].

Real-time performance varies drastically depending on model complexity and hardware. Gibbs et al. reported a significant disparity in processing speeds on a microcontroller: while their activity recognition model inferred in 26 ms, the more complex stress model required 3642 ms (over 3.6 s) per inference [58]. Subathra et al. reported an inference time of 2.0754 s for their Bi-LSTM model [56]. Abdelfattah et al. further illustrated the computational disparity between model families, though their reported times reflected combined training and testing cycles rather than isolated inference latency. RNNs required up to 614 s compared to 0.24 s for Gaussian Naïve Bayes and 37.2 s for XGBoost (the best classifier/metric in phase II of the study) [50]. For real-time deployment, inference latency is the critical bottleneck, as training can be performed offline on more powerful hardware.

### 7.2. Standardization of Datasets: The Over-Reliance on WESAD

The WESAD dataset has become the default benchmark for validating multimodal stress models [15,19,20,21,30,32,33,36,50,54,55,59,60,64,65]. WESAD is a controlled laboratory dataset that records synchronized multimodal physiological signals (including EDA, ECG, respiration, skin temperature, and accelerometry) under three well-defined affective conditions: affective neutral, affective stress, and affective amusement. As a result, when studies report stress classification performance using WESAD, the reported outcomes are derived specifically from discrimination involving the affective stress condition relative to neutral or amusement baselines. While WESAD provides a high-quality dataset for laboratory-based research, many studies explicitly acknowledge the constraints of reliance on this exclusive dataset [19]. This creates a strong generalization gap, as WESAD’s demographic is primarily composed of young healthy university students, often with a significant gender imbalance (12 males and 3 females) [33]. Moreover, lab-induced stress (e.g., the Trier Social Stress Test) does not always mirror the noisy, unstructured stress encountered in free-living environments, where motion artifacts and environmental variability can easily break lab-trained pipelines [44]. Research must therefore prioritize the development of more diverse and robust datasets that capture stress across broader populations and contexts, to ensure that models are truly subject-independent and suitable for real-world deployment [54,55].

### 7.3. Real-Time Edge Deployment: Moving from Offline Analysis to On-Chip Inference

To address user privacy concerns and reduce latency, the next generation of stress-sensing technology is moving towards on-device (Edge AI) inference [56,64]. Frameworks like TinyStressNet demonstrate the feasibility of creating specialized classification networks in the kilobyte range (68.17 kB and 49.4 kB for the two models in the study), which allows for sophisticated DL processing without the need of GPU-enabled cloud systems [64]. In parallel, the development of custom wearable hardware, such as electronic bands that utilize STM32 microcontrollers, allows researchers to perform real-time Bi-LSTM directly on the wrist [56]. “Hearable” and wearable platforms increasingly utilize context-aware gating, where accelerometry is used to identify periods of excessive motion before invoking high-power models [58]. Gibbs et al. encountered memory constraints when deploying models on edge devices, reporting the need to reduce their architecture from four convolutional layers to two due to high memory usage (97%) on the Arduino Nano 33 Sense (ARM Cortex-M4, 256 KB SRAM, 1 MB Flash). This reduction decreased model size by 67%, but resulted in a 0.076 drop in accuracy (from 0.956 with the four-layer model). By shifting signal processing and feature extraction from servers to the wearable’s on-device hardware, the field is increasingly bridging the gap between clinical-grade diagnostic capability and real-time wellness monitoring [56,64]. Furthermore, while Subathra et al. developed a wearable band using the STM32F103C8T6 microcontroller, the hardware was primarily dedicated to signal acquisition and cleaning (using Savitzky-Golay filters), with the computationally intensive Bi-LSTM processing offloaded to external environments due to limited onboard resources [56].

## 8. Conclusions

This review confirms that a shift from unimodal systems to multimodal systems shows strong advances in detection of SNS arousal and stress detection. Electrodermal activity remains an “anchor” due to its direct link to sympathetic arousal, but is now being complemented with many modalities covering broad ranges of the human condition. Multimodal systems can consistently outperform unimodal systems [4,45] by capturing the “brain–body” interplay that single signals can miss. HRV has shown to be a strong addition to these systems.

The literature also shows a migration from statistical and rule-based approaches to signal processing to deep learning and automated feature learning via CNNs, LSTMs, and more [34,60]. Modern architectures, such as CNN-LSTM hybrids and Transformers, have proven superior in identifying temporal patterns and textures in physiological signals that once required complex mathematical transformations [34,41,60].

The most persistent challenge identified has come in the form of subject independence. Many models can achieve near-perfect accuracy in controlled laboratory settings under subject-dependent evaluation, but the transition to free-living environments has yet to be shown in many cases, although it has not gone unnoticed [13]. The ability for models to be robust and adaptable to individual differences is the path forward for many researchers. Future success can be seen in domain adaptation and meta-learning frameworks that can extrapolate a generic model to a new user’s unique physiological fingerprint with minimal bootstrapping [13].

Lastly, as research begins to transition to real-world deployment, cost-awareness and computational complexity begin to dominate, with the goal to maximize prediction confidence while minimizing the energy cost of multimodal data acquisition [38]. The development of TinyStressNet and MC-QSDNN models demonstrate that high-fidelity stress inference is now possible within the kilobyte range, paving the way for real-time monitoring that does not depend on cloud connectivity [21,64]. Ultimately, the future of the field belongs to resource-scalable, context-aware architectures, systems that can intelligently switch between ‘budget’ unimodal monitoring and ‘high-fidelity’ multimodal classification to preserve both battery life and diagnostic confidence [38].

## Figures and Tables

**Table 1:** Boolean search criteria used to identify relevant literature on multimodal sympathetic nervous system arousal detection integrating electrodermal activity (EDA). Each line was concatenated with the rest using the AND function.

Search Criteria
(“Electrodermal Activity” OR “EDA” OR “Galvanic Skin Response” OR “GSR”)
(“Multimodal” OR “Multi-sensor” OR “Sensor Fusion” OR “Data Fusion” OR “Photoplethysmography” OR “PPG” OR “Heart Rate Variability” OR “HRV” OR “Skin Temperature” OR “SpO_2_”)
(“Stress” OR “Cognitive Load” OR “Arousal” OR “Affective Computing”)
(“Machine Learning” OR “Deep Learning” OR “Statistical Modeling”)

**Table 2:** Summary of included studies. Modality codes: E—EDA, P—PPG, H—HRV, T—Temp, R = Resp, A—Accel, C—ECG, M—EMG, G—EEG, O—SpO_2_, I—IBI (Inter-Beat Intervals).

Study	Modalities	Fusion	Primary Model	Primary Result
van Lier et al. (2020) [14]	E, P, H, C	Compared	Statistical (Bland-Altman)	Bland-Altman agreement: HR 0.94, SD 0.97, RMSSD 0.97 within limits; EDA signal r = 0.25 (not valid)
Holder et al. (2022) [15]	E, P, T, A	Compared	CNN	Accuracy: WESAD ACC Unimodal: 0.9587 vs. Multimodal BVP/EDA: ~0.79
Radhika et al. (2022) [16]	E, C	Compared	CRNN-SE + Autoencoder	Accuracy: 0.9623
Eldien et al. (2024) [17]	E, P, H, T, A	Compared	Ensemble (LSTM + Transformer + HART)	F1-Score: 0.6716
Das et al. (2018) [18]	E, P, G	Data	Random Forest	F-score (mean): 0.69
Lee et al. Explain (2024) [19]	E, P, T, R, A	Data	Attention-augmented DNN	Accuracy (all modalities): 0.9657
S et al. (2024) [20]	E, T	Data	SNN + ANN + BNN	Accuracy: 0.8864 (Multimodal SNN)
B S et al. (2025) [21]	E, T, C	Data	SNN (MCLeaky)	Accuracy: 0.988
Singh et al. (2011) [22]	E, P, H	Feature	Statistical tracking (TTV)	Detection Rate: 0.82
Kalimeri & Saitis (2016) [23]	E, G	Feature	Random Forest	AUROC (weighted): EEG—0.773 (0.4), EDA—0.533 (1.0), Fusion—0.793 (0.3)
Chen et al. (2017) [24]	E, H, R, A, C	Feature	SVM	Accuracy: 0.999 (inter-drive), 0.897 (cross-drive)
Sano et al. (2018) [12]	E, T, A, Phone patterns	Feature	Not specified	Accuracy: 0.783 (on Stress)
Bianco et al. (2019) [25]	E, P, R, Perinasal Persp.	Feature	Stacked Ensemble (kNN + SVM + ANN)	Micro Accuracy (5-fold): 0.7725
Cipresso et al. (2019) [2]	E, P, H, R	Feature	Logistic Regression	AUC: 0.808
Kalinkov et al. (2019) [26]	E, P, H, C	Feature	SVM (polynomial)	Average classification accuracy (weighted): Audio-video SG1 (ECG + GSR) adaptive: 0.948
Can et al. (2020) [27]	E, H, R, A, C, I	Feature	SVM	Accuracy: 0.99 (per-drive), 0.89 (cross-drive)
Lima et al. (2020) [1]	E, P, H	Feature	Multiple classifiers compared	Accuracy (tri-modal): Emotion recognition: 0.988; Stress detection: 0.991
Rodrigues et al. (2020) [28]	E, H, C, M	Feature	SVM + DT + RF + GNB	Result values: Not Reported
Romine et al. (2020) [29]	E, P, T	Feature	Multiple classifiers compared	Study 1: RF AUC = 0.99, F1 = 0.94 (liberal); AUC = 0.93, F1 = 0.85 (conservative)
Simons et al. (2020) [30]	E, T	Feature	SVM	Accuracy: (1.0 for same-sensor RespiBAN, 0.99 for same-sensor E4)
Arsalan and Majid (2021) [31]	E, P, H, G	Feature	SVM	Accuracy: 0.9625
Garg et al. (2021) [32]	E, T, R, C, M	Feature	Random Forest	F1-score (Random Forest): 0.8334
Iqbal et al. (2021) [33]	E, H, R, C, M, Self Reported	Feature	Logistic Regression	Accuracy: 0.8571 (All modalities)
Lee et al. Driving (2021) [34]	E, P	Feature	Multimodal CNN	Accuracy: 0.9567 (for 30 s signals)
Markova et al. (2021) [35]	E, P, H	Feature	SVM (polynomial)	Accuracy (person-independent, normalized features): 0.9968
Meneses et al. (2021) [36]	E, T	Feature	DCNN	Accuracy (Stress Campaign): 0.98
Askari et al. (2022) [37]	E, P, H, T, A, C, INFR	Feature	LSTM	Balanced Accuracy: 0.9999
Momeni et al. (2022) [38]	E, P, H, R, C	Feature	CAFS framework	Accuracy (best on unseen data): 0.9098
Rahman et al. (2022) [39]	P, H, T, A	Feature	Random Forest	Accuracy: 0.8052
Saha et al. (2022) [40]	E, P	Feature	Random Forest	Accuracy: 0.9213 (RF)
Li et al. Pilot (2023) [41]	E, H, T, R, C, M	Feature	CNN + Transformer	Accuracy: 0.9328 (2-class); 0.8875 (3-class); 0.8485 (4-class)
Li et al. Trans. (2023) [42]	E, P, H, C	Feature	Classical ML (unspecified)	Accuracy: ~0.80 (HRV baseline only)
Pauzi et al. (2023) [43]	E, P, T	Feature	Bootstrapping Ensemble	Accuracy: 0.9582
Sah et al. (2023) [44]	E, P, H, T, A	Feature	CNN	Accuracy: 0.99
Al Fawwaz et al. (2024) [45]	E, P, H, Self-reported load	Feature	SVM (RBF)	Accuracy: 0.8197
Halder et al. (2024) [46]	E, P, T, A	Feature	LSTM	Accuracy: 0.9178 on test
Le Tran Thuan et al. (2024) [47]	E, T	Feature	SVM	F1-score: 0.8
Mathur et al. (2024) [48]	E, P, H	Feature	Decision Tree	Weighted F1 (multimodal): 0.99
Nechyporenko et al. (2024) [49]	E, P, H	Feature	kNN	Accuracy: 0.833
Abdelfattah et al. (2025) [50]	E, P, T, R, A, C, M	Feature	XGBoost	F1-score: 0.998
Boffet et al. (2025) [51]	E, H, A, C	Feature	K-means + LMM	LMM β (EDA_TVSYMP_ → NASA-TLX): 13.80 (p<0.001); k-means between_SS/total_SS: 0.603
Fujii et al. (2025) [52]	E, P, H, T, R, A	Feature	Linear mixed-effects model (LMM) per feature	Significance (*p*-values): All selected features significant (e.g., HRmin *p* = 5.03 $\times \;10^{14}$; RESPmax *p* = 1.63 $\times \;10^{14}$)
İğde et al. (2025) [53]	E, P, H, T, R	Feature	XGBoost	Accuracy: 0.98
M. and K. (2025) [54]	E, P, T, A	Feature	Multigate-LSTM	MAPE: 0.172
Mihirette et al. (2025) [55]	E, H, T, C	Feature	RF + CORAL	F1 score (target after CORAL + RF): 0.62
Subathra et al. (2025) [56]	E, P, HR, I	Feature	Bi-LSTM	Accuracy: 0.9938
Mozafari et al. (2021) [57]	E, P, R, O	Decision	Stacked fusion + DA	Accuracy: 0.767
Gibbs et al. (2024) [58]	E, P, A	Decision	CNN	Accuracy: 0.88
Zhang et al. (2024) [59]	E, P	Decision	BCSA Network	Accuracy: AvgPool ~0.72, Unimodal ~0.57
Gjoreski et al. (2016) [4]	E, P, H, T, A, Context (Activity levels)	Hybrid	ML Ensemble	Accuracy: 0.92 (Real life data)
Naga Pawan et al. (2025) [60]	E, H, R, C	Hybrid	CNN-LSTM	Accuracy: 0.96
Simic et al. (2025) [61]	E, P, H, T, O, Self-reported questionnaires	Hybrid	SVM	MSE: 0.08
Radhika & Oruganti (2021) [62]	E, C	Hybrid	CNN	Accuracy: 0.85 (Depth 4 CNN; CLAS EDA + ECG with transfer learning from ASCERTAIN)
Lyu et al. (2015) [63]	P, H	Unimodal	Signal-processing index	Significance (*p*-values): $\eta^{2}$ = 0.754 (period effect); *p* = 0.019 (difficulty effect)
Akbar et al. (2019) [3]	E, P, R, C, Thermal (PP)	Unimodal	paired *t*-test Δ≠0	*p*-value (Δ from baseline): PP: *p* < 0.001 across 5/6 tasks; 97% subjects Δ > 0 (presentation)
Nkurikiyeyezu (2019) [13]	E, H, C	Unimodal	Decision Forest	Out-of-sample MAE (HRV model): 10.37
Jaiswal et al. (2024) [64]	E	Unimodal	NAS-generated CNN (TinyStress)	Accuracy: 0.8598 (TinyStress2)
Abe and Jung (2025) [65]	E, T, A	Unimodal	Transformer	Macro F1 (best single-modality): 0.7359 ± 0.1923

## Data Availability

No new data were created or analyzed in this study. Date sharing is not applicable.

## Abbreviations

The following abbreviations are used in this manuscript:

EDA	Electrodermal Activity
GSR	Galvanic Skin Response
HRV	Heart Rate Variability
PPG	Photoplethysmography
SKT	Skin Temperature
TEMP	Body Temperature
SpO2	Blood Oxygen Saturation
ECG	Electrocardiogram/Electrocardiography
EMG	Electromyogram/Electromyography
EEG	Electroencephalogram/Electroencephalography
RESP	Respiration/Respiration Rate
ACC	Accelerometry/3-axis Acceleration
BVP	Blood Volume Pulse
IBI	Interbeat Interval
SCL	Skin Conductance Level
SCR	Skin Conductance Response
RRI	R-peak to R-peak interval
SNS	Sympathetic Nervous System
PNS	Parasympathetic Nervous System
AI	Artificial Intelligence
ML	Machine Learning
DL	Deep Learning
XAI	Explainable Artificial Intelligence
CNN	Convolutional Neural Network
RNN	Recurrent Neural Network
LSTM	Long Short-Term Memory
Bi-LSTM	Bidirectional Long Short-Term Memory
ANN	Artificial Neural Network
SNN	Spiking Neural Network
MLP	Multilayer Perceptron
SVM	Support Vector Machine
KNN	K-Nearest Neighbors
RF	Random Forest
XGBoost	Extreme Gradient Boosting
NAS	Neural Architecture Search
TinyML	Tiny Machine Learning
VFCDM	Variable Frequency Complex Demodulation
RMSSD	Root Mean Square of Successive Differences
WPT	Wavelet Packet Transform
HF	High Frequency
EDATVSYMP	Electrodermal Activity Time-Varying Sympathetic Index via VFCDM method

## References

[1] Lima R., Osório D.N., Gamboa H. (2020). Heart Rate Variability and Electrodermal Activity Biosignal Processing: Predicting the Autonomous Nervous System Response in Mental Stress. Communications in Computer and Information Science.

[2] Cipresso P., Colombo D., Riva G. (2019). Computational psychometrics using psychophysiological measures for the assessment of acute mental stress. Sensors.

[3] Akbar F., Mark G., Pavlidis I., Gutierrez-Osuna R. (2019). An Empirical Study Comparing Unobtrusive Physiological Sensors for Stress Detection in Computer Work. Sensors.

[4] Gjoreski M., Gjoreski H., Luštrek M., Gams M. Continuous stress detection using a wrist device: In laboratory and real life. Proceedings of the 2016 ACM International Joint Conference on Pervasive and Ubiquitous Computing: Adjunct.

[5] Boucsein W. (2012). Electrodermal Activity.

[6] Obrist P.A. (1981). Cardiovascular Psychophysiology.

[7] Thayer J.F., Åhs F., Fredrikson M., Sollers J.J., Wager T.D. (2012). A meta-analysis of heart rate variability and neuroimaging studies: Implications for heart rate variability as a marker of stress and health. Neurosci. Biobehav. Rev..

[8] Cacioppo J.T., Tassinary L.G., Berntson G. (2007). Handbook of Psychophysiology.

[9] Healey J., Picard R. (2005). Detecting Stress During Real-World Driving Tasks Using Physiological Sensors. IEEE Trans. Intell. Transp. Syst..

[10] Kreibig S.D. (2010). Autonomic nervous system activity in emotion: A review. Biol. Psychol..

[11] Barrett L.F. (2016). The theory of constructed emotion: An active inference account of interoception and categorization. Soc. Cogn. Affect. Neurosci..

[12] Sano A., Taylor S., McHill A.W., Phillips A.J., Barger L.K., Klerman E.B., Picard R.W. (2018). Identifying objective physiological markers and modifiable behaviors for self-reported stress and mental health status using wearable sensors and mobile phones: Observational study. J. Med. Internet Res..

[13] Nkurikiyeyezu K.N., Yokokubo A., Lopez G.F. Importance of individual differences in physiological-based stress recognition models. Proceedings of the 2019 15th International Conference on Intelligent Environments (IE).

[14] van Lier H.G., Pieterse M.E., Garde A., Postel M.G., de Haan H.A., Vollenbroek-Hutten M.M.R., Schraagen J.M., Noordzij M.L. (2020). A standardized validity assessment protocol for physiological signals from wearable technology: Methodological underpinnings and an application to the E4 biosensor. Behav. Res. Methods.

[15] Holder R., Sah R.K., Cleveland M., Ghasemzadeh H. Comparing the Predictability of Sensor Modalities to Detect Stress from Wearable Sensor Data. Proceedings of the 2022 IEEE 19th Annual Consumer Communications & Networking Conference (CCNC).

[16] Radhika K., Subramanian R., Oruganti V.R.M. (2022). Joint Modality Features in Frequency Domain for Stress Detection. IEEE Access.

[17] Eldien N.A.S., Ali R.E., Ezzeldin M., Zaher M. Unveiling Stress: A Comparative Analysis of Multimodal Sensor Fusion Techniques for Predictive Modeling. Proceedings of the 2024 Intelligent Methods, Systems, and Applications (IMSA).

[18] Das D., Datta S., Bhattacharjee T., Dutta Choudhury A.D., Pal A. Eliminating Individual Bias to Improve Stress Detection from Multimodal Physiological Data. Proceedings of the 2018 40th Annual International Conference of the IEEE Engineering in Medicine and Biology Society (EMBC).

[19] Lee H., Chang J., Jaewon K., Han B., Park S.M. (2024). Developing an Explainable Deep Neural Network for Stress Detection Using Biosignals and Human-Engineered Features. https://ssrn.com/abstract=4881618.

[20] B S A., Rao M., K P.P. Neuromorphic Energy Efficient Stress Detection System using Spiking Neural Network. Proceedings of the 2024 IEEE International Symposium on Circuits and Systems (ISCAS).

[21] B S A., K P.P., Rao M. (2025). MC-QDSNN: Quantized Deep Evolutionary SNN with Multidendritic Compartment Neurons for Stress Detection Using Physiological Signals. IEEE Trans. Comput.-Aided Des. Integr. Circuits Syst..

[22] Singh R.R., Conjeti S., Banerjee R. An approach for real-time stress-trend detection using physiological signals in wearable computing systems for automotive drivers. Proceedings of the 2011 14th International IEEE Conference on Intelligent Transportation Systems (ITSC).

[23] Kalimeri K., Saitis C. Exploring multimodal biosignal features for stress detection during indoor mobility. Proceedings of the ICMI ’16: International Conference on Multimodal Interaction.

[24] Chen L., Zhao Y., Ye P., Zhang J., Zou J. (2017). Detecting driving stress in physiological signals based on multimodal feature analysis and kernel classifiers. Expert Syst. Appl..

[25] Bianco S., Napoletano P., Schettini R. Multimodal car driver stress recognition. Proceedings of the PervasiveHealth’19: The 13th International Conference on Pervasive Computing Technologies for Healthcare.

[26] Kalinkov K.B., Ganchev T.D., Markova V.I. Adaptive Feature Selection through Fisher Discriminant Ratio. Proceedings of the 2019 International Conference on Biomedical Innovations and Applications (BIA).

[27] Can Y.S., Chalabianloo N., Ekiz D., Fernández-Álvarez J., Repetto C., Riva G., Iles-Smith H., Ersoy C. (2020). Real-Life Stress Level Monitoring Using Smart Bands in the Light of Contextual Information. IEEE Sens. J..

[28] Rodrigues C., Fröhlich W.R., Jabroski A.G., Rigo S.J., Rodrigues A.K., Kern de Castro E.K. (2020). Evaluating a New Approach to Data Fusion in Wearable Physiological Sensors for Stress Monitoring. Intelligent Systems.

[29] Romine W.L., Schroeder N.L., Graft J., Yang F., Sadeghi R., Zabihimayvan M., Kadariya D., Banerjee T. (2020). Using Machine Learning to Train a Wearable Device for Measuring Students’ Cognitive Load during Problem-Solving Activities Based on Electrodermal Activity, Body Temperature, and Heart Rate: Development of a Cognitive Load Tracker for Both Personal and Classroom Use. Sensors.

[30] Simons A., Doyle T., Musson D., Reilly J. Impact of Physiological Sensor Variance on Machine Learning Algorithms. Proceedings of the 2020 IEEE International Conference on Systems, Man, and Cybernetics (SMC).

[31] Arsalan A., Majid M. (2021). Human stress classification during public speaking using physiological signals. Comput. Biol. Med..

[32] Garg P., Santhosh J., Dengel A., Ishimaru S. Stress Detection by Machine Learning and Wearable Sensors. Proceedings of the IUI ’21: 26th International Conference on Intelligent User Interfaces.

[33] Iqbal T., Redon-Lurbe P., Simpkin A.J., Elahi A., Ganly S., Wijns W., Shahzad A. (2021). A Sensitivity Analysis of Biophysiological Responses of Stress for Wearable Sensors in Connected Health. IEEE Access.

[34] Lee J., Lee H., Shin M. (2021). Driving Stress Detection Using Multimodal Convolutional Neural Networks with Nonlinear Representation of Short-Term Physiological Signals. Sensors.

[35] Markova V.I., Ganchev T.D., Kalinkov K.B., Markov M. (2021). Detection of acute stress caused by cognitive tasks based on physiological signals. Bull. Electr. Eng. Inform..

[36] Meneses J., Miranda O., Sanchez I., Alvarez C., Rodriguez P. (2021). Design and validation of a pilot stress monitoring system based on a deep convolutional neural network model. Proceedings of the 2021 IEEE/AIAA 40th Digital Avionics Systems Conference (DASC).

[37] Askari M.R., Abdel-Latif M., Rashid M., Sevil M., Cinar A. (2022). Detection and Classification of Unannounced Physical Activities and Acute Psychological Stress Events for Interventions in Diabetes Treatment. Algorithms.

[38] Momeni N., Valdes A.A., Rodrigues J., Sandi C., Atienza D. (2022). CAFS: Cost-Aware Features Selection Method for Multimodal Stress Monitoring on Wearable Devices. IEEE Trans. Biomed. Eng..

[39] Rahman M.M., Xu X., Nathan V., Ahmed T., Ahmed M.Y., McCaffrey D., Kuang J., Cowell T., Moore J., Mendes W.B. Detecting Physiological Responses Using Multimodal Earbud Sensors. Proceedings of the 2022 44th Annual International Conference of the IEEE Engineering in Medicine & Biology Society (EMBC).

[40] Saha S., Jindal K., Shakti D., Tewary S., Sardana V. (2022). Chirplet transform-based machine-learning approach towards classification of cognitive state change using galvanic skin response and photoplethysmography signals. Expert Syst..

[41] Li Y., Li K., Chen J., Wang S., Lu H., Wen D. (2023). Pilot Stress Detection Through Physiological Signals Using a Transformer-Based Deep Learning Model. IEEE Sens. J..

[42] Li Z., Xing Y., Pi Y., Jiang M., Zhang L. (2023). A novel physiological feature selection method for emotional stress assessment based on emotional state transition. Front. Neurosci..

[43] Pauzi T.M.A.A.T.M., Samah A.A., Dela Cruz J.C., Ghaffa D., Nordin R., Abdullah N.F. Classification of Stress using Machine Learning Based on Physiological and Psychological Data from Wearables. Proceedings of the 2023 IEEE 15th International Conference on Humanoid, Nanotechnology, Information Technology, Communication and Control, Environment, and Management (HNICEM).

[44] Sah R.K., Cleveland M.J., Ghasemzadeh H. (2023). Stress Monitoring in Free-Living Environments. IEEE J. Biomed. Health Inform..

[45] Al Fawwaz A., Rahma O.N., Ain K., Ittaqillah S.I., Chai R. (2024). Measurement of Mental Workload Using Heart Rate Variability and Electrodermal Activity. IEEE Access.

[46] Halder N., Setu J.H., Rafid L., Islam A., Amin M.A. Smartwatch-Based Human Stress Diagnosis Utilizing Physiological Signals and LSTM-Driven Machine Intelligence. Proceedings of the 2024 Advances in Science and Engineering Technology International Conferences (ASET).

[47] Le Tran Thuan T., Nguyen P.K., Gia Q.N., Tran A.T., Le Q.K. Machine Learning Algorithms for Stress Level Analysis Based on Skin Surface Temperature and Skin Conductance. Proceedings of the 2024 IEEE 6th Eurasia Conference on Biomedical Engineering, Healthcare and Sustainability (ECBIOS).

[48] Mathur A., Shikha, Sethia D. Body Sensor-Based Multimodal Nurse Stress Detection Using Machine Learning. Proceedings of the 2024 16th International Conference on COMmunication Systems & NETworkS (COMSNETS).

[49] Nechyporenko A., Frohme M., Strelchuk Y., Omelchenko V., Gargin V., Ishchenko L., Alekseeva V. (2024). Galvanic Skin Response and Photoplethysmography for Stress Recognition Using Machine Learning and Wearable Sensors. Appl. Sci..

[50] Abdelfattah E., Joshi S., Tiwari S. (2025). Machine and Deep Learning Models for Stress Detection Using Multimodal Physiological Data. IEEE Access.

[51] Boffet A., Arsac L.M., Ibanez V., Sauvet F., Deschodt-Arsac V. (2025). Detection of Cognitive Load Modulation by EDA and HRV. Sensors.

[52] Fujii H., Vargo A., Iwata M., Kise K. Evaluating In-situ Cognitive Load Using Physiological Data. Proceedings of the AHs 2025: The Augmented Humans International Conference.

[53] İğde M., Kuruoğlu E., Barkana D.E., Uzun I. Use of Machine-Learning Methods to Develop Stress Detection Model. Proceedings of the 2025 10th International Conference on Computer Science and Engineering (UBMK).

[54] Meenalakshmi M., Valarmathi K. (2025). Multigate Long Short-Term Memory-Based Stress Detection from Multimodal Signal. Cogn. Comput..

[55] Mihirette S., Cal E.A.d.l., Tan Q., Sedano J. (2025). Cross-contextual stress prediction: Simple methodology for comparing features and sample domain adaptation techniques in vital sign analysis. Appl. Intell..

[56] Subathra P., Malarvizhi S., Ferents Koni Jiavana K., Patil S. (2025). A Wearable Electronic Band for Stress Understanding Using Machine Learning. IEEE Sens. J..

[57] Mozafari M., Goubran R., Green J.R. A Fusion Model for Cross-Subject Stress Level Detection Based on Transfer Learning. Proceedings of the 2021 IEEE Sensors Applications Symposium (SAS).

[58] Gibbs M., Woodward K., Kanjo E. (2024). Combining Multiple Tiny Machine Learning Models for Multimodal Context-Aware Stress Recognition on Constrained Microcontrollers. IEEE Micro.

[59] Zhang X., Wei X., Zhou Z., Zhao Q., Zhang S., Yang Y., Li R., Hu B. (2024). Dynamic Alignment and Fusion of Multimodal Physiological Patterns for Stress Recognition. IEEE Trans. Affect. Comput..

[60] Naga Pawan Y., Kalime S., Kandimalla P.C.R., Thirupathi V., Rao A., Sravanthi J. Multi-Scale Temporal Feature Learning for Stress State Prediction using CNN-LSTM on WESAD Physiological Signals. Proceedings of the 2025 6th International Conference on Electronics and Sustainable Communication Systems (ICESC).

[61] Simic M., Yammanuru S.R., Saguiafin G., Velvelicham J., Krishnan S. (2025). A Wrist System for Daily Stress Monitoring Using Mid-Level Physiological Fusion and Late Fusion with Survey-Based Labels. Sensors.

[62] Radhika K., Oruganti V.R.M. Stress Detection using CNN Fusion. Proceedings of the 2021 IEEE Region 10 Conference (TENCON 2021).

[63] Lyu Y., Luo X., Zhou J., Yu C., Miao C., Wang T., Shi Y., Kameyama K. Measuring photoplethysmogram-based stress-induced vascular response index to assess cognitive load and stress. Proceedings of the CHI ’15: CHI Conference on Human Factors in Computing Systems.

[64] Jaiswal D., Mukhopadhyay S., Sharma V. TinyStressNet: On-device Stress Assessment with Wearable Sensors on Edge Devices. Proceedings of the 2024 IEEE International Conference on Pervasive Computing and Communications Workshops and other Affiliated Events (PerCom Workshops).

[65] Abe T., Jung Y.A. Modality-Aware Transformer Models for Generalizable Stress Detection. Proceedings of the 2025 International Conference on Platform Technology and Service (PlatCon).

[66] Posada-Quintero H.F., Chon K.H. (2020). Innovations in Electrodermal Activity Data Collection and Signal Processing: A Systematic Review. Sensors.

[67] Benedek M., Kaernbach C. (2010). A continuous measure of phasic electrodermal activity. J. Neurosci. Methods.

[68] Kounios J., Beeman M. (2014). The cognitive neuroscience of insight. Annu. Rev. Psychol..

